# Pharmacogenetics of Vascular Risk Factors in Alzheimer’s Disease

**DOI:** 10.3390/jpm8010003

**Published:** 2018-01-03

**Authors:** Ramón Cacabelos, Arun Meyyazhagan, Juan C. Carril, Pablo Cacabelos, Óscar Teijido

**Affiliations:** 1 EuroEspes Biomedical Research Center, Institute of Medical Science and Genomic Medicine, Bergondo, 15165 La Coruña, Spain; cytogenetics@euroespes.com (A.M.); genomica@euroespes.com (J.C.C.); asistentedireccion@euroespes.com (P.C.); epigenetica@euroespes.com (Ó.T.); 2 Chair of Genomic Medicine, Continental University Medical School, Huancayo 12000, Peru

**Keywords:** Alzheimer’s disease, anti-dementia drugs, APOE, atorvastatin, cholesterol, CYP haplotypes, Enalapril, hypertension, LipoEsar, pharmacogenetics

## Abstract

Alzheimer’s disease (AD) is a polygenic/complex disorder in which genomic, epigenomic, cerebrovascular, metabolic, and environmental factors converge to define a progressive neurodegenerative phenotype. Pharmacogenetics is a major determinant of therapeutic outcome in AD. Different categories of genes are potentially involved in the pharmacogenetic network responsible for drug efficacy and safety, including pathogenic, mechanistic, metabolic, transporter, and pleiotropic genes. However, most drugs exert pleiotropic effects that are promiscuously regulated for different gene products. Only 20% of the Caucasian population are extensive metabolizers for tetragenic haplotypes integrating *CYP2D6-CYP2C19-CYP2C9-CYP3A4/5* variants. Patients harboring CYP-related poor (PM) and/or ultra-rapid (UM) geno-phenotypes display more irregular profiles in drug metabolism than extensive (EM) or intermediate (IM) metabolizers. Among 111 pentagenic (*APOE-APOB-APOC3-CETP-LPL*) haplotypes associated with lipid metabolism, carriers of the H26 haplotype (23-TT-CG-AG-CC) exhibit the lowest cholesterol levels, and patients with the H104 haplotype (44-CC-CC-AA-CC) are severely hypercholesterolemic. Furthermore, *APOE*, *NOS3*, *ACE*, *AGT*, and *CYP* variants influence the therapeutic response to hypotensive drugs in AD patients with hypertension. Consequently, the implementation of pharmacogenetic procedures may optimize therapeutics in AD patients under polypharmacy regimes for the treatment of concomitant vascular disorders.

## 1. Introduction

Alzheimer’s disease (AD) is a complex disorder in which genomic, epigenomic, cerebrovascular, metabolic, and environmental factors are potentially involved [1,2,3,4,5]. About 85% of patients over 75 years of age exhibit an important cerebrovascular component in their neuropathological phenotype [4,5]. Furthermore, AD patients present concomitant disorders including hypertension (20–30%), overweightness or obesity (20–40%), diabetes (20–25%), hypercholesterolemia (>40%), anemia (>20%), metabolic deficiencies (>15%), atherosclerosis (>60%), cardiovascular disease (>40%), and cerebrovascular damage (60%), which require additional treatments [6,7,8]. Most of these clinical conditions and biochemical anomalies show gender differences and may contribute to accelerating the dementia process. The pharmacological treatment of these concomitant pathologies adds complexity and risks to the multifactorial therapeutic intervention employed in patients with dementia. Of major relevance is the treatment of diabetes, hypertension, dyslipidemia, and cardiovascular, cerebrovascular, and neuropsychiatric disorders. AD patients receive a higher burden of psychotropic drugs, especially neuroleptics, antidepressants, and benzodiazepines, than their age-matched counterparts [9]. The chronic treatment of diverse illnesses with pleiotropic drugs increases the risk of drug interactions and toxicity, aggravating the clinical condition of the demented patient. In this context, the incorporation of pharmacogenetic protocols into clinical practice is fundamental to minimize drug–drug interactions and adverse drug reactions (ADRs) and to optimize the global therapeutic outcome, avoiding deleterious effects on mental function and cognition [2,3,6,7,8,10,11].

Major determinants of therapeutic outcome in AD include age- and sex-related factors, pathogenic phenotype, concomitant disorders, treatment modality and polypharmacy, and pharmacogenetics. Different categories of genes are potentially involved in the pharmacogenetic network responsible for drug efficacy and safety. Pathogenic, mechanistic, metabolic, transporter, and pleiotropic genes represent the major genetic determinants of response to treatment in AD [2,6,8,10]. By-products of these genes are integrated in transcriptomic, proteomic, and metabolic networks, which are disrupted in AD and represent potential targets for therapeutic intervention [8,10,11].

AD patients may take over ten different drugs/day for the treatment of dementia-related symptoms (memory deterioration, behavioral changes, and functional decline) and concomitant disorders (cardiovascular, cerebrovascular, parkinsonian, epileptic, hypertensive, dyslipidemic, and metabolic problems). The older the patient, the higher the number of drugs taken in polypharmacy regimes [9]. Approximately 30–40% of current drugs may contribute to abnormal amyloidogenesis [12]. The co-administration of several drugs may cause ADRs, which in some cases require hospitalization. The prevalence of potentially inappropriate medication (PIM) is around 50% in some European cohorts. Cerebral vasodilators are the most widely used class of PIM, accounting for 24.0% of all prescriptions, followed by atropinic drugs and long half-life benzodiazepines. Atropinic drugs are associated with cholinesterase inhibitors in 16% of patients. In over 20% of the patients, behavioral deterioration and psychomotor function can be severely altered by polypharmacy [13]. The principal causes of these iatrogenic effects are the inappropriate combination of drugs, and the genomic background of the patient, responsible for his/her pharmacogenomic outcome.

The cerebrovascular component of AD is a contributing factor to aggravate disease progression [4,5]; cerebrovascular atherosclerosis may coexist with AD [14]; and, in late-onset AD, vascular risk factors are associated with earlier clinical manifestation [15]. Furthermore, the co-administration of drugs for the treatment of vascular risk factors present in AD patients increases the ADR rate and exerts deleterious effects on brain function and cognition. Typical examples of this are heart disorders, hypercholesterolemia, and hypertension. Some drugs for the treatment of concomitant cardio/cerebrovascular disorders may interact with conventional anti-dementia drugs. At the present time, the only way to minimize the impact of potential ADRs in AD patients under polypharmacy protocols is the implementation of pharmacogenetic protocols [16,17].

## 2. Genetic Determinants of the Pharmacogenomic Network

Pharmacogenomics accounts for 60–90% variability in the pharmacokinetics and pharmacodynamics of common drugs in the market. The modest effect (and ADRs) of current AD drugs are in part due to their pharmacogenomic profile, since over 80% of AD patients are deficient metabolizers [3,8,10,17]. The genes involved in the pharmacogenomic response to drugs in dementia fall into five major categories: pathogenic genes associated with AD-related neurodegeneration (*APP*, *PSEN1*, *PSEN2*, *MAPT*, *APOE*, and >600 susceptibility genes), mechanistic genes associated with the mechanisms of action of drugs (receptors, enzymes), metabolic genes encoding enzymes involved in Phase I and Phase II metabolizing reactions in liver and other tissues [Cytochrome P450 (CYPs), UDP Glucuronosyl Transferases (UGTs), and N-Acetyl Transferases (NATs)], transporter genes encoding transporter proteins [ATP-Binding Cassette Transporters (ABCs) and Solute Carrier Transporters (SLCs)], and pleiotropic genes with multi-locative effects in different pathogenic cascades [3,6,8,10,16,17,18,19,20].

Although the *APP*, *PSEN1*, *PSEN2*, and *MAPT* genes are considered major pathogenic genes for AD and classic tauopathies [18,19,20], mutations in these genes represent less than 5% of the AD population; consequently, their influence on AD pharmacogenetics associated with conventional anti-dementia drugs is quantitatively negligible; not so in the case of immunotherapy or secretase inhibitors/modulators addressing amyloid-β (Aβ) deposition. In this case, gene mutations affect the amyloidogenic and/or tauopathic phenotypes and, consequently, the outcome of pharmacological interventions may be affected by particular genotypes. Most anti-AD vaccines (active and passive immunization) are based on transgenic models with *APP*, *PSEN1*, and *PSEN2* mutants [21,22]. Depending on the transgenic model, the phenotypic expression of Aβ deposition may vary and the therapeutic effects of immunization may be different [23].

To date, the most influential gene in AD pharmacogenetics is the *APOE* gene [2,6,7,8,10,16,17,24]. The vast majority of pharmacogenetic studies in AD have been performed with susceptibility genes (*APOE*) and metabolic genes (CYPs) [8,10,24,25]. In general terms, *APOE-3* carriers tend to be the best responders to conventional antidementia drugs (donepezil, rivastigmine, galantamine, and memantine), and *APOE-4* carriers are the worst responders to different treatments [6,7,8,10,14,17,24,25]. The association of the *TOMM40-L/L* genotype with the *APOE-4/4* genotype yields a haplotype (4/4-L/L) that is responsible for early onset of the disease, a faster cognitive decline, and a poor response to treatment [7,8,16,17]. *CYP2D6* variants also influence the therapeutic outcome, with extensive metabolizers as the best responders, followed by intermediate metabolizers; whereas poor and ultra-rapid metabolizers exhibit a deficient response to drugs in terms of efficacy and safety [6,10,16,17,24,25,26]. Those CYP2D6 extensive metabolizers (EMs) who harbor an *APOE-4/4* genotype are poor responders to conventional treatments, reflecting the negative influence that the *APOE-4* allele exerts on the pharmacogenetic outcome in AD patients [6,10,16,17,24,25,26].

Other recent pharmacogenetic studies with pathogenic or mechanistic genes indicate that the response to cholinesterase inhibitors (AChEIs) can be modulated by genes associated with the cholinergic system. Genetic variants in *CHRNA7*, encoding a subunit of the acetylcholine receptor (α7-nAChR), are associated with the response to AChEIs. Pharmacogenetic studies in an Italian cohort with two single nucleotide polymorphisms (SNPs) (rs6494223 and rs8024987) in the *CHRNA7* gene revealed that the rs6494223 variant may affect response to AChEIs [27]. Variability in the clinical response to AChEIs is also associated with 2 SNPs in the intronic region of *CHAT* rs2177370 and rs3793790 [28]. The *CHRNA7* T allele (rs6494223) also associates with a better response to AChEIs, and there is further confirmation that *APOE*-*4* carriers are the worst responders to conventional AChEIs [29]. Butyrylcholinesterase (BChE) activity increases with disease progression and may replace acetylcholinesterase function. The BChE K-variant is associated with lower acetylcholine-hydrolyzing activity and with a poor response to donepezil, similar to that observed in *APOE-4* carriers [30]. A genome-wide association study in 176 AD patients identified 2 SNPs with apparent response to treatment; one SNP (rs6720975A) maps in the intronic region of *PRKCE*, and the another one (rs17798800A) is an intergenic segment acting as a cis-regulator of *NBEA* [31]. Old studies identified SNPs in Phase II reactions enzymes, such as gluthatione *S*-transferase theta, to be associated with the hepatotoxic effects of tacrine [32]. Many other variants influence the therapeutic response to AChEIs and combination treatments in AD [2,3,7,8,10,16,17,24,25,26].

All these genes involved in the pharmacogenomic network are under the influence of the epigenetic machinery (DNA methylation, histone/chromatin modifications, microRNA (miRNA) regulation) conditioning their expression and the efficiency of their drug-metabolizing products (enzymes, transporters) [26,33,34].

## 3. Drug Metabolism

Over 70% of AD patients are deficient metabolizers for the *CYP2D6/2C19/2C9* trigenic cluster; and for the *CYP2D6/2C19/2C9/3A4* tetragenic cluster, more than 80% of the patients exhibit a deficient metabolizer geno-phenotype [3,17]. These four *CYP* genes encode enzymes responsible for the metabolism of 60–80% of drugs of current use, showing ontogenic-, age-, sex-, circadian- and ethnic-related differences [10,24,35,36]. CYP2D6 enzymes metabolize over 900 different drugs (371 substrates, 300 inhibitors, and 18 inducers). CYP2C9 enzymes metabolize over 600 drugs (311 substrates, 375 inhibitors, and 41 inducers). Nearly 500 drugs are metabolized via CYP2C19 enzymes (281 substrates, 263 inhibitors, and 23 inducers). CYP3A4 and 3A5 enzymes metabolize over 1900 drugs (1033 substrates, 696 inhibitors, and 241 inducers) [36].

The distribution and frequency of *CYP2D6* genotypes are very similar in the general population (GP) and in AD, with the exception of the *CYP2D6*-*3/*4 genotype, which is absent in AD samples [17]. In the GP, CYP2D6 extensive metabolizers (EMs) account for 58.85%, whereas intermediate metabolizers (IMs) account for 31.11%, poor metabolizers (PMs) 4.49%, and ultra-rapid metabolizers (UMs) 5.55% [8,10,17]. In AD, EMs, IMs, PMs, and UMs represent 57.54%, 31.01%, 5.49%, and 5.96%, respectively [17]. There is an accumulation of AD-related genes of risk in PMs and UMs. EMs and IMs are the best responders, and PMs and UMs are the worst responders to a combination therapy with AChEIs, neuroprotectants, and vasoactive substances [2,10,37]. The pharmacogenetic response in AD appears to be dependent upon the networking activity of genes involved in drug metabolism and genes associated with AD pathogenesis [2,6,10,16,17,38]. By phenotypes, in the GP, CYP2C9-PMs represent 4.82%, IMs 33.83%, and EMs 61.35%. In AD, PMs, IMs, and EMs represent 4.76%, 34.87%, and 60.37%, respectively [8,10,17]. The frequencies of the *CYP2C19* geno-phenotypes in the GP are *CYP2C19-*EMs (74.11%), *CYP2C19*-IMs (24.43%), and *CYP2C19*-PMs (1.46%) [17]. EMs, IMs, and PMs account for 75.41%, 23.56%, and 1.03%, respectively, in AD [8,10,16,17]. Concerning *CYP3A4/5* polymorphisms in AD, 83.84% of the cases are EMs (CYP3A5*3/*3), 14.62% are IMs (CYP3A5*1/*3), and 1.54% are RMs (CYP3A5*1/*1), whereas, in the GP, EMs, IMs, and RMs represent 82.17%, 16.48%, and 1.35%, respectively [17].

**Figure 1:**
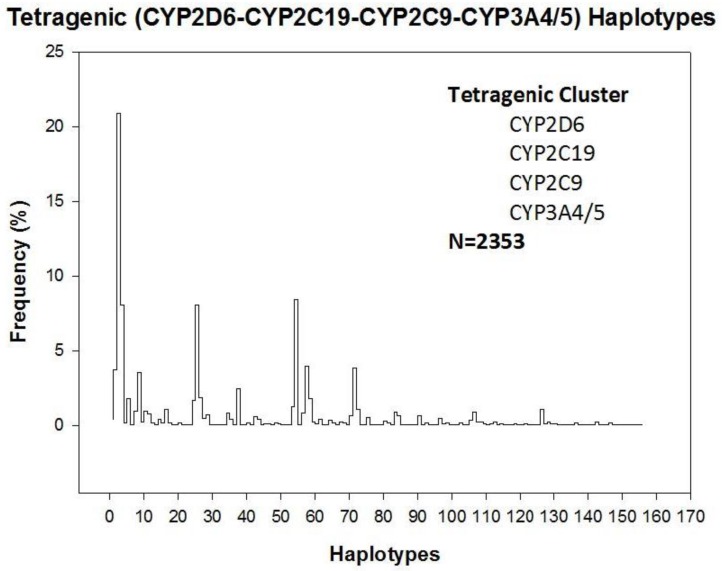
Tetragenic (*CYP2D6-CYP2C19-CYP2C9-CYP3A4/5*) haplotypes in the Spanish population.

**Figure 2:**
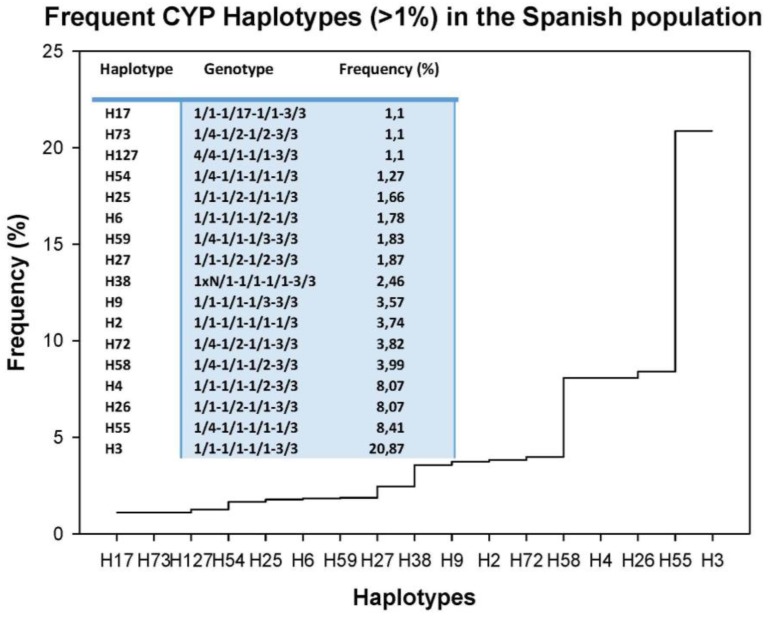
Frequent tetragenic *CYP2D6-CYP2C19-CYP2C9-CYP3A4/5* haplotypes (>1%) in the Spanish population.

Tetragenic haplotypes integrating *CYP2D6*, *CYP2C9*, *CYP2C19*, and *CYP3A4/5* variants yield 156 genotypes (Figure 1). The most frequent haplotype is H3 (1/1-1/1-1/1-3/3) (20.87%), representing full extensive metabolizers, and only 17 haplotypes exhibit a frequency higher than 1% in the Iberian population (Figure 2). In addition to H3, the most frequent haplotypes (>2%) are H55 (1/4-1/1-1/1-1/3) (8.41%), H26 (1/1-1/2-1/1-3/3) (8.07%), H4 (1/1-1/1-1/2-3/3) (8.07%), H58 (1/4-1/1-1/2-3/3) (3.99%), H72 (1/4-1/2-1/1-3/3) (3.82%), H2 (1/1-1/1-1/1-1/3) (3.74%), H9 (1/1-1/1-1/3-3/3) (3.57%), and H38 (1xN/1-1/1-1/1-3/3) (2.46%) (Figure 2). Among Caucasians, about 80% of the population is deficient for the biotransformation of current drugs metabolized via CYP2D6-2C9-2C19-3A4 enzymes [17].

## 4. Genetic Determinants of Lipid Metabolism and Vascular Function

Among hundreds of genes potentially involved in AD pathogenesis and concomitant disorders (cardiovascular and cerebrovascular disorders, hypercholesterolemia, and hypertension), at least four categories of genes deserve special attention: (i) genes associated with lipid metabolism (*APOB*, *APOC3*, *APOE*, *CETP*, and *LPL*), (ii) genes associated with endothelial function and hypertension (*NOS3*, *ACE*, and *AGT*); (iii) genes associated with immune function and inflammation (*IL1B*, *IL6*, *IL6R*, and *TNFA*); and (iv) genes associated with thrombosis and coagulation (*F2*, *F5*, and *MTHFR*) [17,36,39].

**Figure 3:**
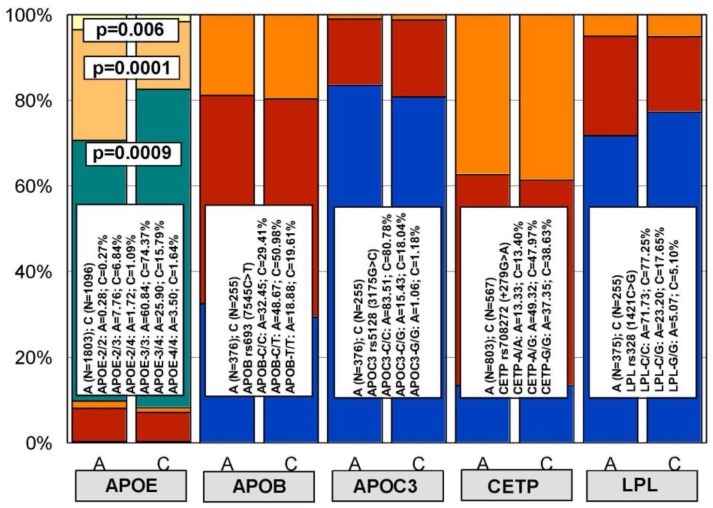
Distribution and frequency of polymorphic variants of the *APOE*, *APOB*, *APOC3*, *CETP,* and *LPL* genes in the general population and in patients with Alzheimer’s disease. A: Alzheimer’s disease; C: Control Population. Color codes: Blue: *APOE-2/2*, *APOB-C/C*, *APOC3-C/C*, *CETP-A/A*, *LPL-C/C*; Red: *APOE-2/3*, *APOB-C/T*, *APOC3-C/G*, *CETP-A/G*, *LPL-C/G*; Orange: *APOE-2/4*, *APOB-T/T*, *APOC3-G/G*, *CETP-G/G*, *LPL-G/G*; Green: *APOE-3/3*; Golden: *APOE-3/4*; Yellow: *APOE-4/4*.

**Figure 4:**
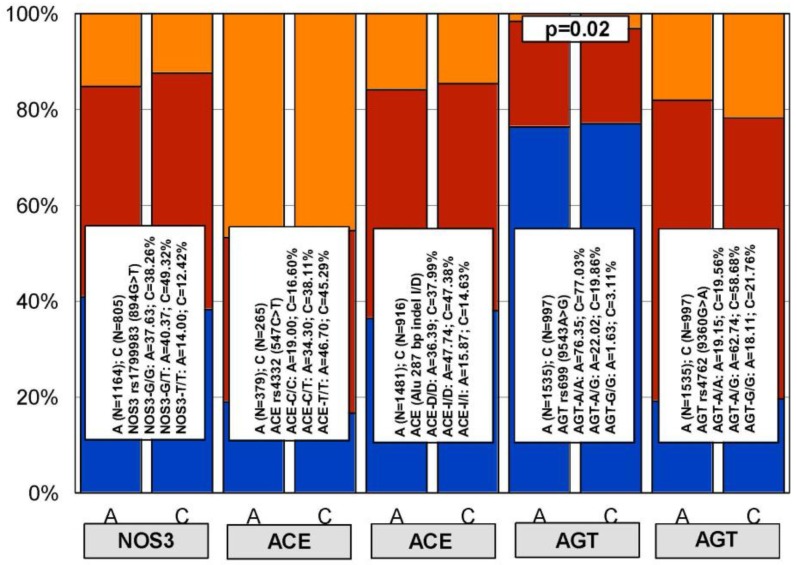
Distribution and frequency of polymorphic variants of the *NOS3*, *ACE*, and *AGT* genes in the general population and in patients with Alzheimer’s disease. Color codes: Blue: *NOS3-G/G*, *ACE-C/C*, *ACE-D/D*, *AGT-A/A*; Red: *NOS3-G/T*, *ACE-C/T*, *ACE-I/D*, *AGT-A/G*; Orange: *NOS3-T/T*, *ACE-C/T*, *ACE-I/I*, *AGT-G/G*.

Although differences in genotype distribution and frequencies of all these genes between patients with AD and control subjects are negligible, except in the case of *APOE* [39] (Figure 3 and Figure 4), some of them may influence the pharmacogenetic outcome in the treatment of major risk factors for dementia, such as hypercholesterolemia, cardiovascular disorders, and hypertension [39,40,41,42,43]. Furthermore, many of these genes interact in pathogenic cascades contributing to alter blood pressure, brain cholesterol, and Aβ metabolism, subsequently accelerating neuronal death in AD. A clear example is the angiotensin-converting enzyme (ACE), which degrades Aβ, and ACE inhibitors that contribute to slow down cognitive decline. In fact, some SNPs in the ACE gene (rs1800764 and rs4291) are associated with cognitive modification and therapeutic response to anti-hypertensive treatment with ACE inhibitors [44].

## 5. Pharmacogenetics of Hypercholesterolemia in Alzheimer’s Disease

Alterations in cholesterol (CHO) metabolism are involved in AD pathogenesis and over 40% of AD patients are hypercholesterolemic. High CHO levels are also associated with vascular dementia [45]. It has been postulated that statins, prescribed as lipid-lowering drugs to patients at risk for cardiovascular conditions, may be beneficial in AD [46,47,48]. Statins are currently used in AD [9]; however, clinical evidence shows conflicting results and poor benefits [49,50,51,52]. The potential beneficial effect of statins and reduction in AD risk varied across statin molecules, sex, and race/ethnicity [53]. Other studies indicate that simvastatin exacerbates amyloid angiopathy [54]. In contrast, lovastatin might reduce Aβ levels in humans [55]. Some mechanisms by which simvastatin and atorvastatin might facilitate amyloid-β-protein degradation would be by increasing neprilysin secretion from astrocytes through activation of Mitogen-activated protein kinase/extracellular signal-regulated kinases (MAPK/Erk1/2) pathways [56], regulation of cholesterol in lipid rafts, suppression of inflammation, and inhibition of oxidative stress [47]. The pleiotropic effects of statins (simvastatin, atorvastatin, cerivastatin, fluvastatin, pravastatin, and rosuvastatin) (Table 1 are apparently APOE-independent; however, APOE is a fundamental factor in the regulation of lipid metabolism, and APOE variants influence the therapeutic effect of most hypolipemic compounds, including statins [8,10,17,36].

Cognitive deterioration shows a clear age-dependent profile in AD, with an average decline of 3–5 points/year (Mini-Mental State Examination (MMSE) score); however, total CHO levels do not appear to affect mental deterioration in AD [17]. Blood lipid levels also show a moderate age-dependent profile. In the GP, CHO levels tend to increase with age, reaching a plateau at 60–70 years of age and declining thereafter; however, CHO levels in AD tend to diminish in an age-related fashion, but in age-matched samples CHO levels tend to be higher in AD as compared to GP [17].

In previous studies, we have demonstrated that *APOE* and *CYP* variants (2D6, 2C9, 2C19, 3A4/5) influence basal CHO levels and the therapeutic response of AD patients to hypolipemic compounds [10,14,17]. In a larger study with 1345 hypercholesterolemic AD patients (CHO > 220 mg/dL), we investigated the pharmacogenetics of cholesterol response to Atorvastatin (10–20 mg/day) plus SardiLipin (E-SAR-94010; LipoEsar^®^; Ebiotec, Bergondo, Spain) (500 mg/day), a nutraceutical compound of the ProteoLipin/LipoFishin family [57], with lipid-lowering effects and anti-atherosclerotic and neuroprotective properties (Patent ID: P9602566) [10,36,58]. In the whole sample, the response rate (RR) was 78.96% responders (CHO < baseline levels) and 21.04% non-responders (CHO ≥ baseline levels) after one month of treatment (Figure 4). APOE-related basal CHO levels are significantly different, with females showing higher CHO levels than males; however, females and males responded similarly to the hypolipemic treatment [17]. The stratification of patients according to their *APOE*, *APOB*, *APOC3*, *CETP*, and *LPL* genotypes showed no genotype-related differences at basal CHO levels, except in the case of *APOE-4* carriers, where the highest baseline levels of CHO were found in *APOE-4/4* carriers [17] (Figure 3).

**Figure 5:**
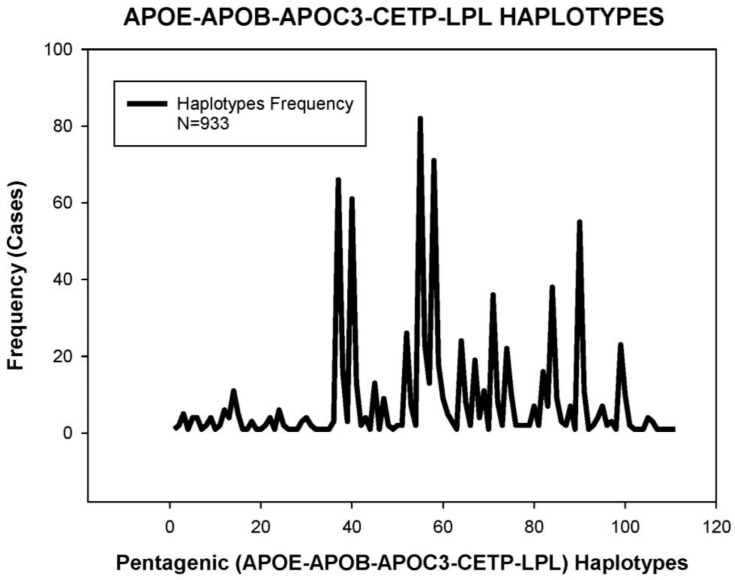
Distribution and frequency of pentagenic haplotypes integrating *APOE*, *APOB*, *APOC3*, *CETP*, and *LPL* genotypes in patients with Alzheimer’s disease.

**Figure 6:**
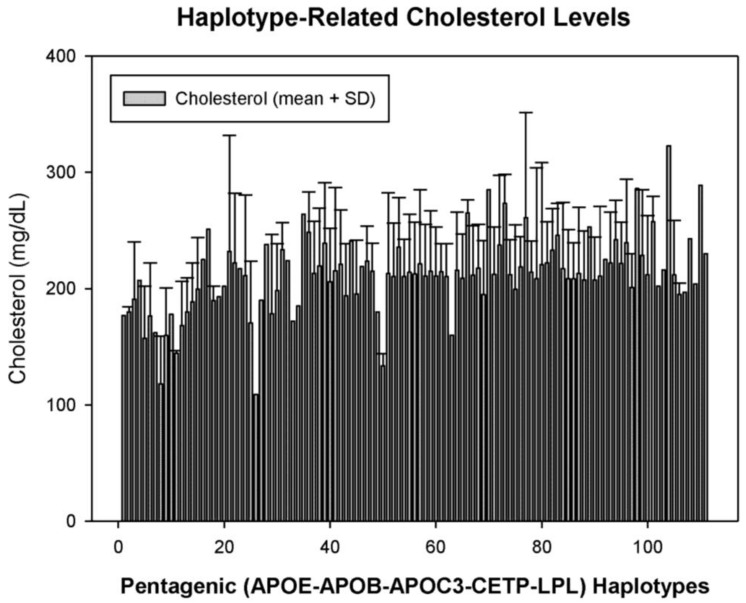
Pentagenic haplotype-related basal cholesterol levels in patients with Alzheimer’s disease.

In a selected group of 933 AD patients, we constructed a pentagenic haplotype integrating all possible variants of the *APOE + APOB + EPOC3 + CETP + LPL* genes and identified 111 haplotypes (H) (Figure 5) with differential basal CHO levels (Figure 6). About 75% of these haplotypes in the AD population have a frequency below 1%, 10% have a frequency between 1% and 2%, 8% have a frequency between 2% and 5%, and only 4% of the haplotypes are present in more than 5% of AD patients [17]. The haplotypes most frequently found are *H55* (33-CT-CC-AG-CC) (8.79%), *H58* (33-CT-CC-GG-CC) and *H37* (33-CC-CC-AG-CC) (7.07%). Haplotypes *H104* (44-CC-CC-AA-CC) (0.11%), *H110* (44-TT-CC-AG-CG) (0.11%) and *H98* (34-TT-CC-AA-CG) (0.11%) showed the highest CHO levels, and the lowest levels corresponded to haplotypes *H26* (23-TT-CG-AG-CC) (0.11%), *H8* (23-CC-CG-AG-CC) (0.21%), *H50* (33-CC-GG-AG-CC) (0.21%), and *H63* (33-CT-CG-AA-GG) (0.11%) [17] (Figure 6).

**Figure 7:**
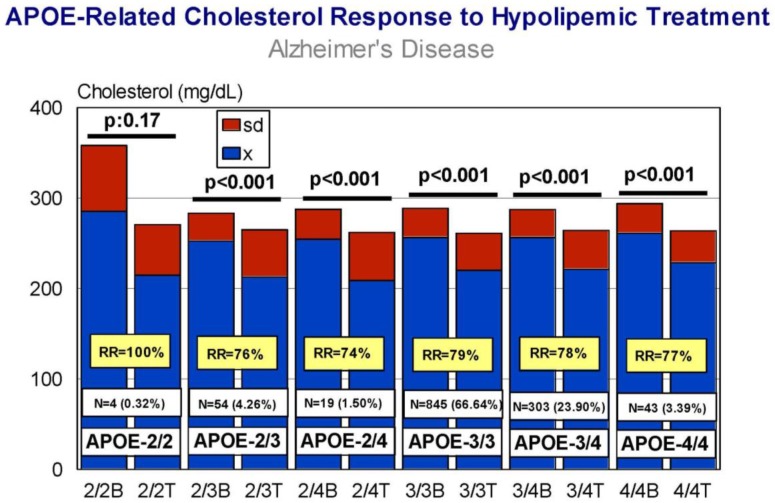
*APOE*-related cholesterol response to a hypolipemic treatment in hypercholesterolemic patients with Alzheimer’s disease. B: Basal values; T: Treatment (Atorvastatin: 10–20 mg/day; LipoEsar: 500 mg/day).

**Figure 8:**
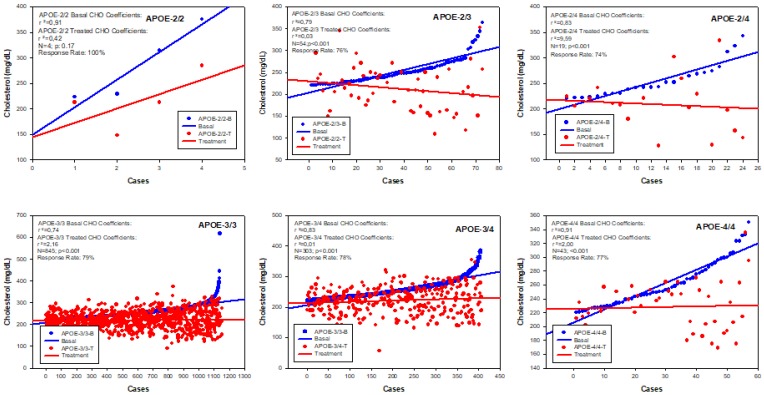
*APOE*-related individual response of cholesterol to a hypolipemic treatment (Atorvastatin + LipoEsar) in hypercholesterolemic patients with Alzheimer’s disease.

The results of *APOE*-related cholesterol response to hypolipemic treatment in hypercholesterolemic AD patients revealed that in absolute terms all *APOE* variants respond similarly (RR > 70%) to treatment, with a significant reduction in CHO levels (*p* < 0.001) (Figure 7); however, genotype-related correlation analysis case by case (Figure 8) and comparative correlation analyses of *APOE* variants show a clear differential *APOE*-related pattern of CHO response to treatment [17].

**Figure 9:**
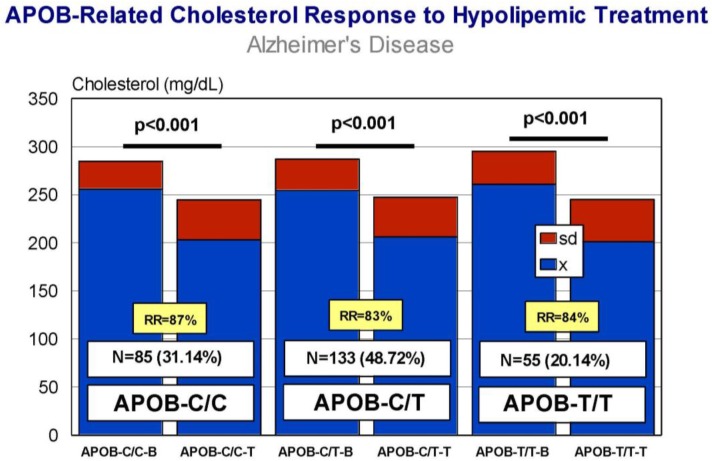
*APOB*-related cholesterol response to a hypolipemic treatment in hypercholesterolemic patients with Alzheimer’s disease (Atorvastatin: 10–20 mg/day; LipoEsar: 500 mg/day).

**Figure 10:**
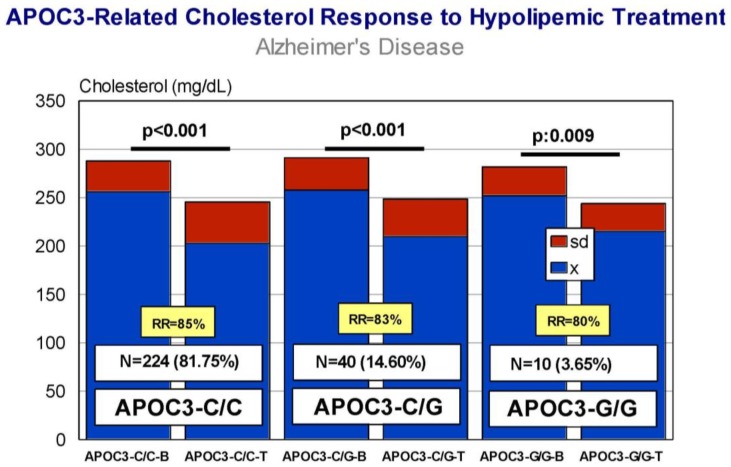
*APOC3*-related cholesterol response to a hypolipemic treatment in hypercholesterolemic patients with Alzheimer’s disease (Atorvastatin: 10–20 mg/day; LipoEsar: 500 mg/day).

**Figure 11:**
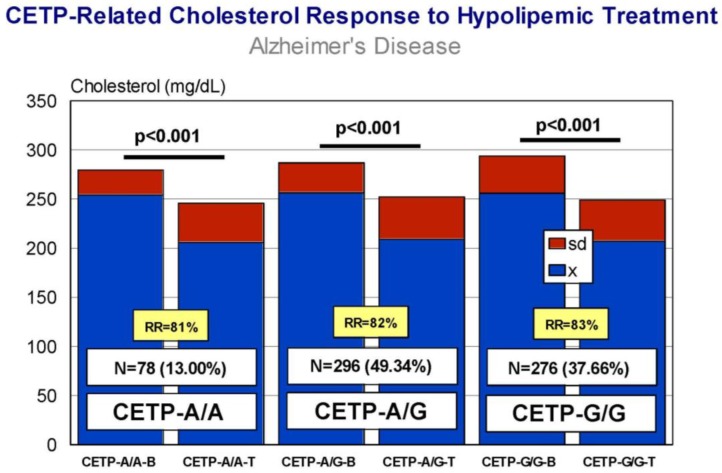
*CETP*-related cholesterol response to a hypolipemic treatment in hypercholesterolemic patients with Alzheimer’s disease (Atorvastatin: 10–20 mg/day; LipoEsar: 500 mg/day).

**Figure 12:**
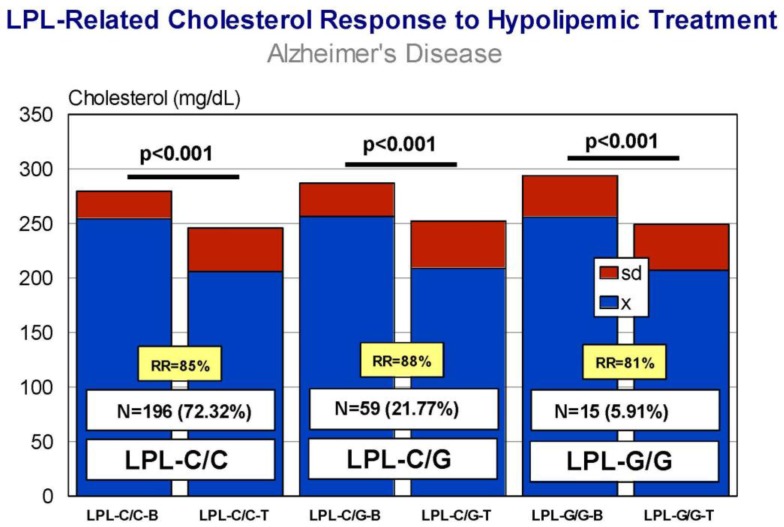
*LPL*-related cholesterol response to a hypolipemic treatment in hypercholesterolemic patients with Alzheimer’s disease (Atorvastatin: 10–20 mg/day; LipoEsar: 500 mg/day).

Carriers of *APOB*-C/C, *APOB*-C/T, and *APOB*-T/T variants exhibit a similar response (RR > 80%), with a significant decrease in CHO levels after treatment (Figure 9) and almost identical efficiency in comparative analyses. *APOC3*-C/C, *APOC3*-C/G, and *APOC3*-G/G carriers also respond similarly (*p* < 0.001) (RR > 80%) (Figure 10), with a differential comparative profile among *APOC3* variants. *CETP*-A/A, *CETP*-A/G, and *CETP*-G/G carriers show an identical response (*p* < 0.001; RR > 80%) (Figure 11), with insignificant variability in comparative studies among *CETP* variants. The same therapeutic response is observed in *LPL*-C/C, *LPL*-C/G, and *LPL*-G/G carriers (*p* < 0.001; RR > 80%) (Figure 12); however, in this case, *LPL*-C/C are the best responders, *LPL*-C/G are intermediate responders, and *LPL*-G/G are the most heterogeneous responders [17].

*CYP* haplotype-related blood total CHO levels are very heterogeneous, but absolute values of total CHO among the most frequent haplotypes are almost identical. The histograms of frequency associated with CHO levels are qualitatively different among carriers of different *CYP* variants. Basal CHO levels are higher in AD patients harboring the *CYP2D6*-*1/*1 and *1xN/*1 genotypes than in the corresponding GP genotypes, but no differences have been found according to the EM, IM, PM, or UM condition. The therapeutic response according to SNPs of metabolic genes (*CYP2D6*, *CYP2C9*, *CYP2C19*, and *CYP3A4/4*) in hypercholesterolemic patients is variable and geno-phenotype-dependent. Although all *CYP2D6* variants exhibit a positive response to treatment, significant differences have only been detected in *2D6*-*1/*1, 2D6-*1/*4, and *2D6*-*1/*6 carriers. In absolute values, CYP2D6 extensive, intermediate, poor, and ultra-rapid metabolizers behave in a similar manner with a significant reduction in CHO levels; however, the RR is different in EMs (81%), IMs (78%), PMs (84%), and UMs (90%), indicating a variable efficiency of CYP2D6 enzymes. The comparative analysis indicates that carriers of mutant enzymes (PMs > UMs), with limitations in drug metabolism, display a more efficient response to hypolipemic treatment [17]. No differences are present in basal CHO levels between the GP and AD patients related to *CYP2C9* genotypes. CYP2C9-EMs, -IMs, and -PMs show a similar response, with lower RR (75%) in PMs as compared with EMs (81%) and IMs (82%), and a clear differential comparative profile. AD cases harboring the CYP2C19-*1/*2 genotype, corresponding to CYP2C19-IMs, exhibit higher basal CHO levels than their homologs in the GP. The CHO response among CYP2C19-EMs, IMs, PMs, and UMs is more variable, with PMs showing a deficient response in comparison to EMs, IMs, and UMs, and a clearly different behavioral profile, especially in PMs and UMs [17]. *CYP3A4/5* geno-phenotypes in AD and GP show similar basal CHO levels. CYP3A4/5-RMs respond poorly to hypolipemic treatment, with the worst RR (66%), whereas CYP3A4/5-EMs and -IMs exhibit an excellent response (*p* < 0.001; RR > 80%) [17].

Most of these effects can, in part, be explained on a pharmacogenetic basis. It is obvious that a simple stratification of patients according to single genotypes is of poor value for a fine interpretation of pharmacogenetic results; however, the integration of gene clusters associated with specific phenotypes yields informative haplotypes with potential utility in pharmacogenetic studies. It is likely that thousands of genes are involved in CHO metabolism, and probably not a single gene plays an absolute dominant role over the others; however, some genes exert a powerful effect on other congeners associated with a specific pathogenic cascade (e.g., *APOE* in AD) or a pharmacogenetic pathway (e.g., *APOE* vs. *CYPs* in AD treatment with donepezil) [3,10,25,36,37]. The lipid-lowering effects and the anti-atherosclerotic properties of LipoEsar are *APOE*-dependent, with *APOE-3* carriers acting as the best responders and *APOE-4* carriers behaving as the worst responders [10,58].

Statins (·-Hydroxy-3-Methylglutaryl-Coenzyme A Reductase (HMGCR) inhibitors) are among the most prescribed drugs worldwide (Table 1). Inhibition of HMGCR results in decreased intrahepatic CHO synthesis, together with the upregulation of LDL-CHO receptors, increased LDL-CHO uptake by hepatocytes, and decreased levels of systemic LDL-CHO. The most relevant ADRs of statins are muscle, liver, and brain toxicity.

Several pathogenic (*ACE, APOA1, APOA5, APOB, APOC3, APOE, CETP, FGB, GNB3, LIPC, MMP3, MTTP, NOS3, PON*) and mechanistic genes (*ABCB1, ABCC1, APOA1, APOA5, APOB, APOC3, APOE, CRP, CYP11B2, HMGCR, IL10, IL6, LDLR, MMP3, PON1, TNF*) influence the effects of atorvastatin. This statin is a major substrate of CYP2C8 and CYP3A4/5; it is a strong inhibitor of CYP2C19, a moderate inhibitor of ABCB1, CYP2B6, CYP2C8, CYP2C9, CYP2C19, CYP2D6, CYP3A4, and HMGCR, and an inducer of CYP2B6 and CYP7A1. Atorvastatin is transported by ABCA1, ABCB1, ABCB11, ABCC1, ABCC2, ABCC3, ABCG2, SLCO1B1, and SLCO1B3 proteins and interacts with the products of various pleiotropic genes (*APOA1, APOE, CRP, CYP11B2, ESR1, GNB3, HTR3B, IL6, IL10, ITGB3, MMP3, TNF*, and *USP5*) [16,36].

*HMGCR* variants (rs17244841, rs3846662, and rs17238540) (H7 haplotype) are responsible for an attenuated lipid-lowering response to statins [59,60]. Sex-related changes in cholesterol response to statins have been reported in carriers of the *HMGCR-AA* genotype at rs3846662, who have higher levels of total and LDL-cholesterol. The percentage reduction in LDL-cholesterol upon statin treatment is decreased in women with the AA genotype compared with women without it. In hypercholesterolemic patients, *HMGCR* alternative splicing may explain 22–55% of the variance in statin response [61].

Several polymorphisms in the *SLCO1B1* gene may alter transport of statins into the liver. The *SLCO1B1* 521C (rs4149056) variant is associated with diminished effects of simvastatin, atorvastatin, lovastatin, and pravastatin [62]. Another transporter that modifies the effects of statins is *ABCB1*, especially in carriers of the *ABCB1*-1236T (rs1128503), 2677T (rs2032582), and 3435T (rs1045642) variants (TTT haplotype) [63]. Other transporters potentially affecting statin transport and metabolism include *ABCC2*, *ABCG2*, *ABCB11*, *SLC15A1*, *SLC22A6*, *SLC22A8*, *SLCO2B1*, *SCLO1B3,* and *SLCO1B3* [59]. Statin metabolism is influenced by the following enzymes: CYP3A4/5, CYP2C8/9, CYP2C19, CYP2D6, UGT1A1, UGT1A3, and UGT2B7 [59]. The deficient CYP3A4 enzyme in CYP3A4*22 (rs35599367) carriers alters the pharmacokinetics and pharmacodynamics of simvastatin, atorvastatin, and lovastatin [64], and the CYP3A5*3 (rs776746) variant (loss-of-function allele) causes a high increase in the bioavailability of simvastatin [65,66]. The powerful effect of atorvastatin in CYP3A4/5-IMs is the result of a poor metabolization of atorvastatin by mutant CYP3A4/5 enzymes, since atorvastatin is a major substrate of CYP3A4/5. In contrast, the lack of effect in CYP3A4/5-RMs results from a rapid destruction of the drug in the liver mediated by excessive CYP3A4/5 enzymatic activity. Therefore, the dose of statins should be adjusted to the metabolizing condition of each patient to optimize the lipid-lowering effects of statins and to avoid toxicity [36,64]. Furthermore, the co-administration of the nutraceutical LipoEsar enhances the hypolipemic effect of atorvastatin and facilitates a dose reduction of the statin by 50%, minimizing potential ADRs in susceptible patients [10,16,17].

## 6. Pharmacogenetics of Hypertension in Alzheimer’s Disease

**Figure 13:**
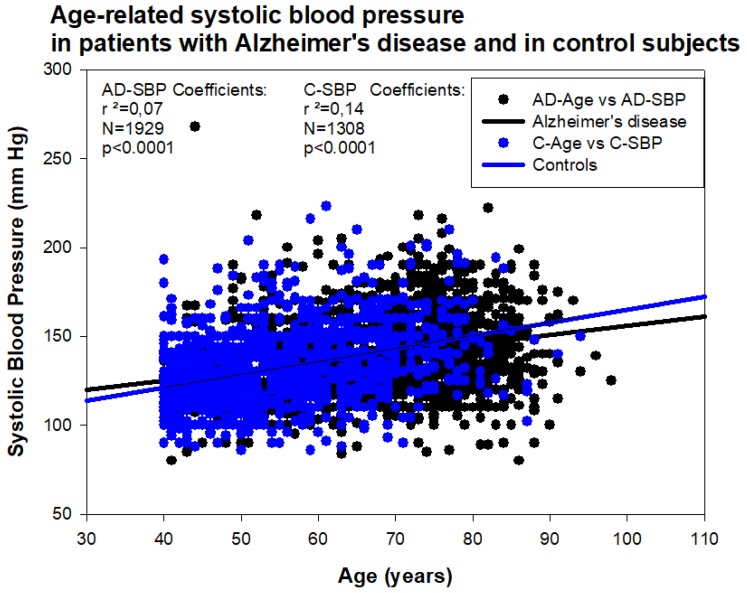
Age-related systolic blood pressure in patients with Alzheimer’s disease and in the control population.

Hypertension is considered a modifiable risk factor for AD, together with diabetes, obesity, physical inactivity, depression, smoking, and low educational attainment [67]. Hypertension is also associated with the worsening of cognitive function, but not with evidence of increased amyloidopathy and/or tauopathy [68]. However, in AD, both hypotension and hypertension may induce deleterious effects on brain function, cognition, and psychomotor praxis. In a sample of 1308 healthy subjects (age: 55.27 ± 10.82 years; range: 40–94; Females: 607; Males: 701) and 1929 AD cases (age: 67.59 ± 11.76 years; range: 40–98; Females: 1175; Males: 754), we found that hypertension is an age-dependent condition (Figure 13). Systolic blood pressure (SBP) in AD (139.44 ± 21.79 mm Hg) tends to be higher than in the control population with no family history of dementia (132.41 ± 21.14 mm Hg) (*p* < 0.001); in contrast, diastolic blood pressure (DBP) does not show differences between both groups. Among AD cases, we found 15.81% of patients with SBP < 120 mm Hg; 57.60% were normotensive (SBP: 120–150 mm Hg), and 26.59% were hypertensive (SBP > 150 mm Hg). Regarding DBP, 9.80% were hypotensive (DBP < 70 mm Hg), 65.47% normotensive (DBP: 70–85 mm Hg), and 24.73% hypertensive (DBP > 85 mm Hg). In the control population, 14.67% showed systolic hypertension and 17.30% diastolic hypertension. Hypertension was almost 50% less frequent in the control population than in the AD cohort (*p* < 0.0001).

In general, over 20% of AD patients are hypertensive and receive different modalities of hypotensive agents, including central α-adrenergic agonists, vasodilators, diuretics, nitrates, nitrites, phosphodiesterase inhibitors, calcium channel blockers, angiotensin-converting enzyme inhibitors, angiotensin II receptor antagonists, mineralcorticoid (aldosterone) receptor antagonists, renin inhibitors, and other plural miscellaneous compounds (Table 2).

**Figure 14:**
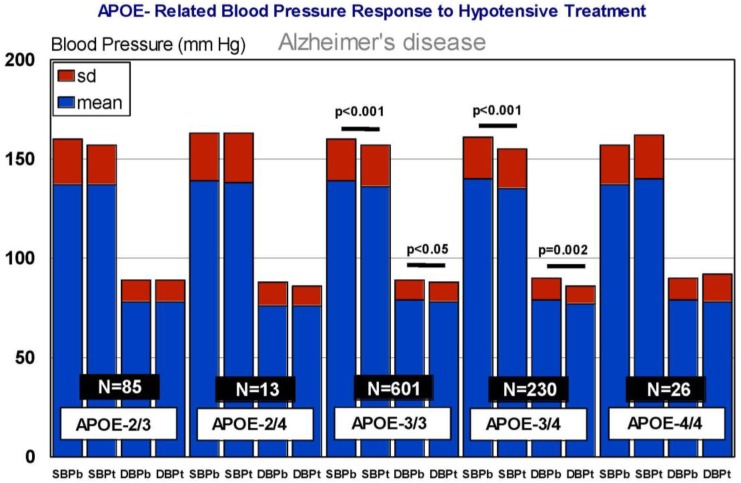
*APOE*-related blood pressure response to a hypotensive treatment. SBPb: Systolic blood pressure. SBPt: Systolic blood pressure (Enalapril, 10–20 mg/day). DBP: Diastolic blood pressure.

**Figure 15:**
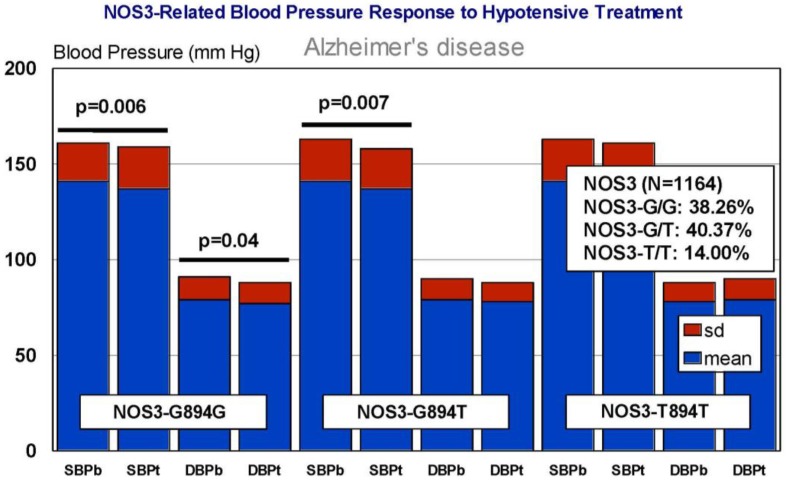
*NOS3*-related blood pressure response to a hypotensive treatment. SBPb: Systolic blood pressure. SBPt: Systolic blood pressure (Enalapril, 10–20 mg/day). DBP: Diastolic blood pressure.

**Figure 16:**
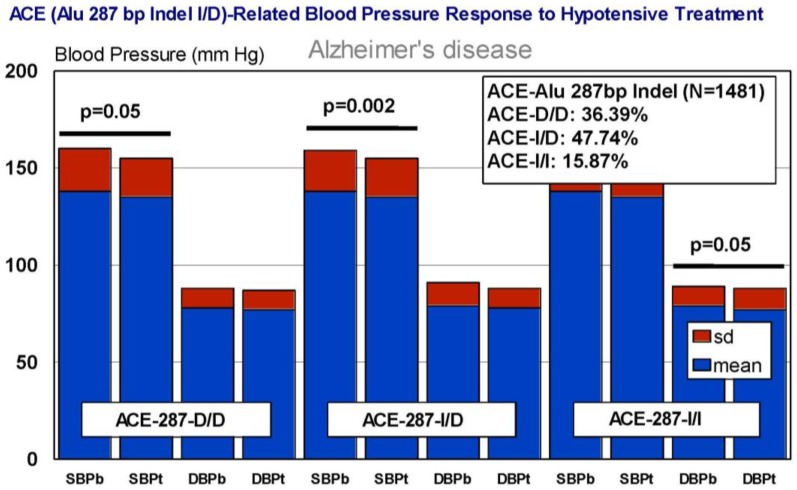
*ACE*-related blood pressure response to a hypotensive treatment. SBPb: Systolic blood pressure. SBPt: Systolic blood pressure (Enalapril, 10–20 mg/day). DBP: Diastolic blood pressure.

**Figure 17:**
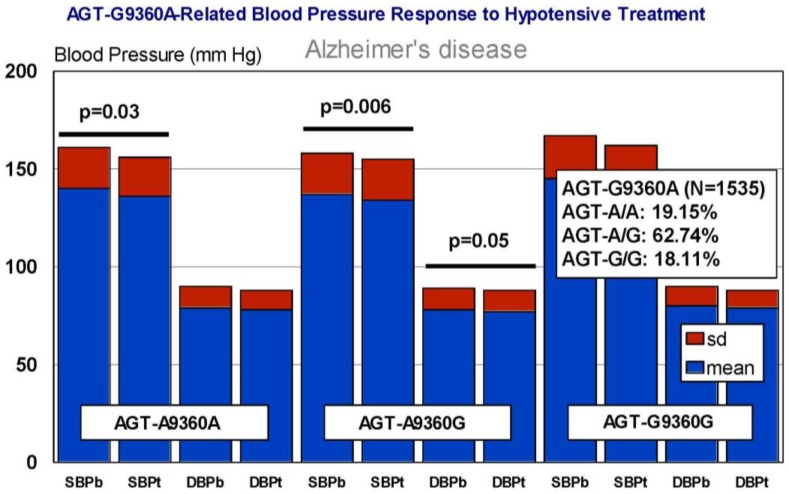
*AGT*-related blood pressure response to a hypotensive treatment. SBPb: Systolic blood pressure. SBPt: Systolic blood pressure (Enalapril, 10–20 mg/day). DBP: Diastolic blood pressure.

In our casuistic, we treated hypertensive AD patients with Enalapril (10–20 mg/day) for one month and performed a pharmacogenetic study assessing the potential influence of *APOE*, *NOS3*, *ACE*, *AGT*, and *CYP2D6*, *2C19*, *2C9*, and *3A4/5* variants on blood pressure response to this competitive inhibitor of the angiotensin-converting enzyme. In AD patients, SBP decreased from 139.44 ± 21.79 to 136.28 ± 21.13 mm Hg (*p* < 0.0001); and DBP decreased from 79.04 ± 11.02 to 77.78 ± 10.64 mm Hg (*p* = 0.004). A similar response was observed in hypertensive non-demented patients. Analysis of the genotype-related blood pressure response to *APOE* (Figure 14), *NOS3* (Figure 15), *ACE* (Figure 16), and *AGT* variants (Figure 17) revealed that specific polymorphisms in these genes differentially influence the hypotensive effect of Enalapril in AD patients. For instance, only *APOE-3/3* and *APOE-3/4* carriers responded with significant reductions in SBP (*p* < 0.001) and DBP values (*p* < 0.05) (Figure 14); and *APOE-4* carriers tended to show higher hypertensive levels than *APOE-4* non-carriers (Figure 14). *NOS3-G/G* carriers responded better than *NOS3-G/T>NOS3-T/T* carriers (Figure 15). Polymorphic variants of the *ACE* rs4332 (547C>T) SNP did not show any effect; however, *ACE-I/D* carriers of the Alu 287 bp Indel I/D exhibited a better response than *ACE-D/D* and *ACE-I/I* carriers in SBP, and *ACE-I/I* carriers responded better in DBP than *ACE-D/D* and *ACE-I/D* (Figure 16). Probably, the clearest response was observed among *AGT-A/A* and *AGT-A/G* carriers, who responded significantly better than *AGT-G/G* carriers (Figure 17). Concerning CYP variants, CYP2D6-, CYP2C19-, and CYP2C9-EMs and -IMs are better responders that PMs or UMs. *CYP3A4/5* variants did not show any effect on blood pressure changes. However, it is very likely that different CYP variants influence basal SBP and DBP values.

Enalapril [l-Proline,1-[*N*-[1-(ethoxycarbonyl)-3-phenylpropyl]-l-alanyl]-,(*S*)-,(*Z*)-2-butenedioate (1:1)] is a competitive ACE inhibitor that prevents the conversion of angiotensin I to angiotensin II, a potent vasoconstrictor, resulting in lower levels of angiotensin II, which causes an increase in plasma renin activity and a reduction in aldosterone secretion [36]. *ACE*, *ADRB2*, *AGT*, *AGTR1*, *BDKRB2*, and *NOS3* are mechanistic genes that regulate the effects of Enalapril. Forty-eight genes show evidence of involvement in blood pressure regulation and 6 new signals of association in or near *HSPB7*, *TNXB*, *LRP12*, *LOC283335*, *SEPT9*, and *AKT2* have been reported, with new replication evidence for *EBF2* and *NFKBIA* [69]. This ACE inhibitor is an apparent substrate of CYP3A4/5 enzymes and is transported by *SLC15A1*, *SLC22A6*, *SLC22A7*, *SLC22A8*, and *SLCO1A2* gene products [36]. Interestingly, we did not find any effect of CYP3A4/5 variants in EM, IM and RMs on blood pressure changes after one month of treatment with Enalapril in AD patients. Therefore, further studies seem to be necessary to elucidate the role of CYP variants in Enalapril metabolism.

Basic and clinical studies suggest that some hypotensive agents may be beneficial in AD [70,71]. Angiotensin-converting enzyme inhibitors (ACEIs) and angiotensin receptor blockers (ARBs) are common anti-hypertensive treatments, but have differential effects on cortical amyloid [70].

The renin angiotensin system (RAS) is directly associated with hypertension, and overactivity of RAS may perpetuate AD-related brain inflammation. It has been reported that Candesartan, an angiotensin type 1 receptor (AT1R) blocker (ARB), may prevent astrocyte and microglial activation and neuroinflammation in the brain of hypertensive rats. This hypotensive agent, by activating Wnt/β-catenin signaling, promotes neurogenesis during the hypertensive state, prevents astrocyte and microglial activation, and improves hippocampal neurogenesis in hypertensive state, independently of its classical hypotensive effect [71]. In humans with mild cognitive impairment (MCI) or AD, the individuals treated with ARBs showed greater hippocampal volumes and brain parenchymal fraction than those treated with ACEIs. ARBs were also associated with significantly better performance in tests of episodic and verbal memory, language, and executive function [70].

## 7. EKG Conditions and Blood Pressure in Alzheimer’s Disease

**Figure 18:**
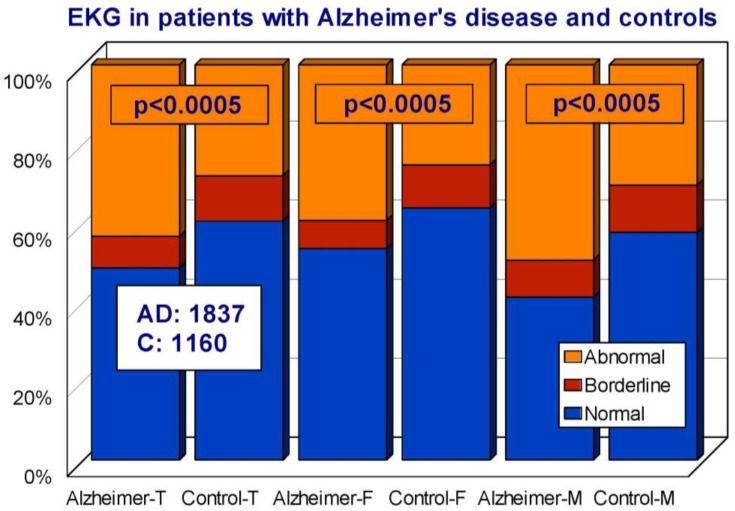
Electrocardiogram (EKG) in patients with Alzheimer’s disease and controls. T: Total; F: Females; M: Males.

**Figure 19:**
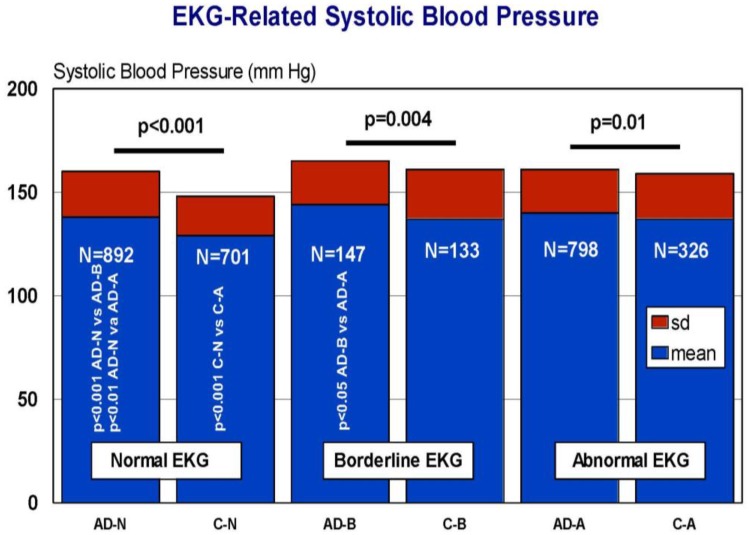
Electrocardiogram (EKG)-related systolic blood pressure in patients with Alzheimer’s disease and controls. AD: Alzheimer’s disease. C: Control. N: Normal. B: Borderline. A: Abnormal. AD-N vs. AD-B, *p* < 0.001; AD-N vs. AD-A, *p* < 0.01; C-N vs. C-A, *p* < 0.001; AD-B vs. AD-A, *p* > 0.05.

Cardiovascular drugs, associated or not with anti-hypertensive agents, are frequently given to elderly subjects and demented patients. Approximately 50% of AD patients show some kind of heart disorder susceptible to pharmacological treatment. In our AD population, we studied the frequency of cardiovascular disorders in comparison with the control population. We divided both cohorts according to a normal, borderline, or abnormal electrocardiogram (EKG). Among AD patients, 48.56% showed a normal EKG, 8.00% a borderline EKG, and 43.44% an abnormal EKG. In the control population, the EKG was normal in 60% of the cases, borderline in 11.47%, and abnormal in 25.33% (Odds ratio (OR) 1.92; 95% Confidence interval (CI) 1.63–2.26; *p* < 0.0005 AD vs. controls) (Figure 18). Interestingly enough, SBP levels, but not DBP levels, were significantly higher in AD than in controls, unrelated to normal, borderline, or abnormal EKGs (Figure 19), probably suggesting that hypertension and heart disease are independent risk factors for AD and vascular dementia [45]. In fact, recent studies indicate that myocardial infarction is associated with a higher risk of dementia [72]; prevalent and incident cardiovascular disorders predict cognitive decline [73]; and a consistent inverse association was observed between systolic blood pressure and AD [74]. However, in our casuistic, SBP tends to be higher in AD as compared to controls with no history of dementia. Furthermore, small-vessel disease-related white matter lesions are associated with gray matter atrophy and with cognitive performance [75]; and hypertension is a contributing factor to vascular microlesions in AD brains.

## 8. Concluding Remarks

According to the present examples and abundant data collected from the international literature [36], it seems clear that cardio-cerebrovascular risk factors, such as blood pressure changes, hypercholesterolemia, and heart disorders may contribute to exacerbating the disease process in AD patients. Most of these medical conditions require pharmacological intervention with drugs that can interact among themselves and with anti-dementia drugs as well. Additionally, the therapeutic response to conventional drugs is genotype-dependent, but routine pharmacogenetic studies are scarcely performed; and over 90% of AD patients simultaneously receive different types of drugs for concomitant disorders with a high risk of drug–drug interactions and ADRs. From a practical perspective, in order to help physicians in their daily clinical practice, the implementation of relatively simple protocols of pharmacogenetics would be of great utility [17]. Examples derived from the present results suggest (i) that the dose of anti-dementia drugs in *APOE-4* carriers and in CYP2D6-PMs and CYP2D6-UMs be adjusted, (ii) that the dose of statins in CYP3A4-IMs be reduced and statins metabolized via CYP enzymes in CYP3A4-RMs avoided due to inefficacy, and (iii) that the dose of ACE inhibitors (or select a more effective anti-hypertensive agent) in APOE-4/4, NOS3-T/T, and GT-G/G carriers be adjusted.

Some reflections might be necessary for improving the multifactorial therapeutic intervention in a complex disorder such as AD: (i) a better characterization of the roles played in drug efficacy and safety by genes involved in the pharmacogenomic network is highly desirable; (ii) since most genes are under the influence of the epigenetic machinery, pharmacoepigenomics is becoming an attractive field that deserves special attention, and some epigenetic drugs might also be helpful in selected AD cases, although at present most epigenetic drugs pose technical problems (bioavailability, toxicity, and brain penetration) [26,34,76]; (iii) drug–drug interactions represent a problematic issue in over 80% of AD patients; to palliate this difficulty, simple pharmacogenetic protocols should be introduced in the clinical setting to help physicians in their daily prescription activity, to minimize ADRs; (iv) since the neurodegenerative process underlying AD neuropathology starts 20–30 years before the onset of the disease, novel therapeutics should be addressed to prevent premature neuronal death (symptomatic drugs have proven to be poorly effective), and a better knowledge of drugs with potential neurodegenerative effects after chronic treatments is also necessary to limit their inappropriate use; (v) specific biomarkers for AD are necessary in three different contexts: predictive markers before disease onset, early diagnosis in initial stages, and drug monitoring (in both preventive and/or therapeutic strategies); and (vi) educational programs are fundamental for physicians to be aware of the usefulness of pharmacogenomics to prescribe more accurately, to avoid adverse reactions and to optimize the limited therapeutic resources available for the treatment of dementia [8,16,17].

## Figures and Tables

**Table 1:** Pharmacological properties and pharmacogenetics of statins.

HMG CoA Reductase Inhibitors		
Drug	Properties	Pharmacogenetics
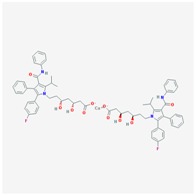	**Name: ATORVASTATIN CALCIUM** **Molecular Formula:** C_66_H_68_CaF_2_N_4_O_10_ **Molecular Weight:** 1155.341726 g/mol **Mechanism:** Inhibits HMG-CoA reductase, resulting in a compensatory increase in the expression of LDL receptors on hepatocyte membranes and a stimulation of LDL catabolism. **Effect:** Anticholesteremic Agent; HMG-CoA Reductase Inhibition; Apolipoprotein B reduction; Triglyceride reduction; Anti- atherosclerotic; Heart-health effects.	**Pathogenic genes:** *ABCA1*, *ACE*, *APOA1*, *APOA5*, *APOB*, *APOC3*, *APOE*, *CETP*, *FGB*, *GNB3*, *LDLR*, *LIPC*, *MMP3*, *MTTP*, *NOS3*, *PON1* **Mechanistic genes:** *ABCB1*, *ABCC1*, *APOA1*, *APOA5*, *APOB*, *APOC3*, *APOE*, *CRP*, *CYP11B2*, *HMGCR*, *IL10*, *IL6*, *LDLR*, *MMP3*, *PON1*, *TNF* **Metabolic genes:** **Substrate:** *CYP2C8*, *CYP3A4* (major), *CYP3A5* **Inhibitor:** *ABCB1*, *CYP2B6* (moderate), *CYP2C8*, *CYP2C9* (moderate), *CYP2C19* (strong), *CYP2D6* (moderate), *CYP3A4* (moderate), *HMGCR* **Inducer:** *CYP2B6*, *CYP7A1* **Transporter genes:** *ABCA1*, *ABCB1*, *ABCB11*, *ABCC1*, *ABCC2*, *ABCC3*, *ABCG2*, *SLCO1B1*, *SLCO1B3* **Pleiotropic genes:** *APOA1*, *APOE*, *CRP*, *CYP11B2*, *ESR1*, *FGB*, *GNB3*, *HTR3B*, *IL6*, *IL10*, *ITGB3*, *MMP3*, *NOS3*, *TNF*, *USP5*
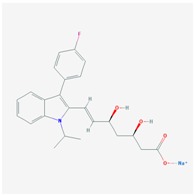	**Name: FLUVASTATIN SODIUM** **Molecular Formula:** C_24_H_25_FNNaO_4_ **Molecular Weight:** 433.447772 g/mol **Mechanism:** Acts by competitively inhibiting HMGCR, the enzyme that catalyzes reduction of HMG-CoA to mevalonate. HDL is increased while total, LDL and VLDL cholesterols, apolipoprotein B, and plasma triglycerides are decreased. **Effect:** Anticholesteremic Agent; HMG-CoA Reductase Inhibition; Heart-health effects; Antineoplastic activity; Immune response modulation	**Pathogenic genes:** *ABCA1*, *ACE*, *APOA1*, *APOA5*, *APOB*, *APOC3*, *APOE*, *CETP*, *CYP7A1*, *LDLR*, *LIPC*, *LPL*, *NOS3*, *PPARD*, *PON1* **Mechanistic genes:** *APOA1*, *APOB*, *HMGCR*, *LPL*, *PON1* **Metabolic genes:** **Substrate:** *CYP1A1* (minor), *CYP2C8* (minor), *CYP2C9* (major), *CYP2D6* (minor), *CYP3A4* (minor), *UGT1A3* **Inhibitor:** *CYP1A2* (weak), *CYP2C8* (weak), *CYP2C9*, (moderate), *CYP2C19* (moderate), *CYP2D6* (moderate), *CYP3A4* (moderate), *HMGCR* **Inducer:** *CYP2B6*, *CYP3A4*, **Transporter genes:** *ABCA1*, *ABCB1*, *ABCB11*, *ABCC2*, *ABCG2*, *SLC15A1*, *SLC22A8*, *SLCO1B1*, *SLCO1B3*, *SLCO2B1* **Pleiotropic genes:** *ACE*, *APOA1*, *APOE*, *NOS3*, *NR1I2*, *NR1I3*, *PPARD*, *USP5*
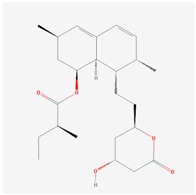	**Name: LOVASTATIN** **Molecular Formula:** C_24_H_36_O_5_ **Molecular Weight:** 404.53964 g/mol **Mechanism:** Acts by competitively inhibiting HMG-CoA reductase, enzyme which catalyzes rate-limiting step in cholesterol biosynthesis. **Effect:** Anticholesteremic Agent; HMG-CoA Reductase Inhibition; Heart-health effects; Antineoplastic activity.	**Pathogenic genes:** *ABCA1*, *APOA1*, *APOA5*, *APOB*, *APOC3*, *CETP*, *LDLR*, *LIPC*, *LPL* **Mechanistic genes:** *APOA1*, *APOB*, *CETP*, *HMGCR*, *LDLR* **Metabolic genes:** **Substrate:** *CYP3A4* (major), *CYP3A5*, *UGT1A3.* **Inhibitor:** *ABCB1*, *CYP2C8*, *CYP2C9* (weak), *CYP2C19* (strong), *CYP2D6* (weak), *CYP3A4* (weak), *HMGCR*, *KCNH2* **LInducer:** *CYP2B6*, *CYP7A1* **Transporter genes:** *ABCA1*, *ABCB1*, *ABCB11*, *ABCC2*, *ABCG2*, *KCNH2*, *SLCO1B1*, *SLCO1B3* **Pleiotropic genes:** *TP53*, *USP5*
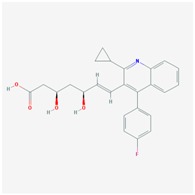	**Name: PITAVASTATIN** **Molecular Formula:** C_25_H_24_FNO_4_ **Molecular Weight:** 421.460763 g/mol **Mechanism:** Works to control the synthesis of cholesterol via competitive inhibition of the liver enzyme, HMG-CoA reductase. As a result, a compensatory increase in LDL-receptor expression can be observed which facilitates an increase LDL catabolism. **Effect:** Anticholesteremic Agent; HMG-CoA Reductase Inhibition; Heart-health effects.	**Pathogenic genes:** *APOB*, *LDLR* **Mechanistic genes:** *APOB*, *HMGC*, *LDLR*, *PPARG*, *PON1*, *VCAM1* **Metabolic genes:** **Substrate:** *ABCB1*, *CYP2C8* (minor), *CYP2C9* (minor), *CYP3A4* (minor), *SLCO1B1*, *UGT1A3*, *UGT2B7.* **Inhibitor:** *HMGCR* **Transporter genes:** *ABCB1*, *ABCG2*, *SLCO1B1*, *SLCO2B1* **Pleiotropic genes:** *PPARG*, *VCAM1*
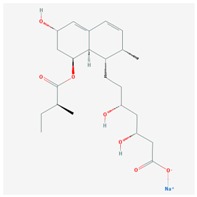	**Name: PRAVASTATIN SODIUM** **Molecular Formula:** C_23_H_35_NaO_7_ **Molecular Weight:** 446.509569 g/mol **Mechanism:** A competitive inhibitor of 3-hydroxy-3-methylglutaryl coenzyme A (HMG-CoA) reductase. **Effect:** Anticholesteremic Agent; HMG-CoA Reductase Inhibition; Heart-health effects; Immune response modulation; MHC II suppression.	**Pathogenic genes:** *ABCA1*, *ACE*, *APOA1*, *APOA5*, *APOB*, *APOC3*, *APOE*, *CETP*, *CYP7A1*, *FGB*, *LDLR*, *LIPC*, *LPL*, *NOS3* **Mechanistic genes:** *APOA1*, *APOB*, *APOC3*, *APOE*, *CRP*, *HMGCR*, *LDLR*, *IL1B*, *IL6*, *IL10*, *MMP2*, *NOS3* **Metabolic genes:** **Substrate:** *ABCB1*, *ABCC2*, *CYP3A4* (minor), *SLCO1B1*, *SLCO2B1*, *UGT1A3* **Inhibitor:** *ABCB1*, *CYP2C8*, *CYP2C9* (weak), *CYP2C19* (weak), *CYP2D6* (weak), *CYP3A4* (weak), *HMGCR* **Inducer:** *CYP1A1*, *CYP2B6*, *CYP2E1* **Transporter genes:** *ABCA1*, *ABCB1*, *ABCB11. ABCG2*, *SLC22A8*, *SLCO1A2*, *SLCO1B1*, *SLCO1B3*, *SLCO2B1* **Pleiotropic genes:** *ACE*, *ALDH1A1*, *APOE*, *CBS*, *FGB*, *HTR3B*, *IL6*, *IL10*, *ITGB3*, *LEP*, *MTHFR*, *MMP2*, *MMP3*, *NOS3*, *TP53*, *USP5*
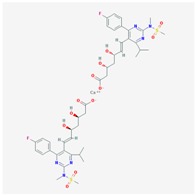	**Name: ROSUVASTATIN CALCIUM** **Molecular Formula:** C_44_H_54_CaF_2_N_6_O_12_S_2_ **Molecular Weight:** 1001.137366 g/mol **Mechanism:** Inhibitor of HMG-CoA reductase, rate-limiting enzyme in cholesterol synthesis. This results in compensatory increase in expression of LDL receptors on hepatocyte membranes and stimulation of LDL catabolism. **Effect:** Anticholesteremic Agent; HMG-CoA Reductase Inhibition; Heart-health effects; Antineoplastic activity.	**Pathogenic genes:** *ABCA1*, *ACE*, *APOA1*, *APOA5*, *APOB*, *APOC3*, *APOE*, *CETP CYP7A1*, *FGB*, *LDLR*, *LIPC*, *LPL*, *NOS3* **Mechanistic genes:** *APOA1*, *APOB*, *CETP*, *FGB*, *HMGCR*, *LDLR*, *LPL*, *NOS3* **Metabolic genes:** **Substrate:** *ABCB1*, *ABCC1*, *ABCC4*, *ABCG2*, *CYP2C9* (minor), *CYP2C19*, *CYP3A4* (minor), *SLC10A1*, *SLCO1A2*, *SLCO1B1*, *SLCO1B3*, *SLCO2B1*, *UGT1A3* **Inhibitor:** *CYP3A4*, *CYP3A5*, *HMGCR*, *SLCO1B1* **Inducer:** *CYP2B6*, *CYP2C8*, *CYP2C9*, *CYP2C19*, *CYP3A4* **Transporter genes:** *ABCA1*, *ABCB1*, *ABCB11*, *ABCG2*, *SLC10A1*, *SLC22A8*, *SLCO1A2*, *SLCO1B1*, *SLCO1B3*, *SLCO2B1* **Pleiotropic genes:** *ACE*, *APOE*, *FGB*, *ITGB3*, *NOS3*, *TCF20*, *USP5*
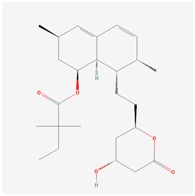	**Name: SIMVASTATIN** **Molecular Formula:** C_25_H_38_O_5_ **Molecular Weight:** 418.56622 g/mol **Mechanism:** Prodrug requiring hydrolysis in vivo for activity. Inhibits HMG-CoA reductase, causing subsequent reduction in hepatic cholesterol synthesis. Reduces serum concentrations of total cholesterol, LDL-C, Apo B, and triglycerides **Effect:** Anticholesteremic Agent; HMG-CoA Reductase Inhibition; Heart-health effects; Anti-inflammatory activity	**Pathogenic genes:** *ABCA1*, *APOA1*, *APOA5*, *APOB*, *APOC3*, *APOE*, *CETP*, *CYP7A1*, *FGB*, *GNB3*, *LIPC*, *LDLR*, *LPL*, *NOS3* **Mechanistic genes:** *ABCA1*, *APOA1*, *APOB*, *APOE*, *CETP*, *HMGCR*, *IL6*, *LDLR*, *LPL*, *VCAM1* **Metabolic genes:** **Substrate:** *ABCB1*, *CYP2C8* (minor), *CYP2C9*, (minor), *CYP2C19* (minor), *CYP2D6*, *CYP3A4* (major), *CYP3A5* ,*POR*, *SLCO1B1*, *UGT1A3* **Inhibitor:** *CYP2C8* (weak), *CYP2C9* (weak), *CYP2C19* (strong), *CYP2D6* (weak), *CYP3A4* (moderate), *HMGCR* **Inducer:** *CYP2B6* **Transporter genes:** *ABCA1*, *ABCB1*, *ABCB11*, *ABCC2*, *ABCC3*, *ABCG2*, *SLCO1B1*, *SLCO1B3* **Pleiotropic genes:** *APOE*, *F2*, *FGB*, *GNB3*, *NOS3*, *PRNP*, *TNF*, *VCAM1*, *USP5*

*ABCA1*: ATP binding cassette subfamily A member 1; *ABCB1*: ATP binding cassette subfamily B member 1; *ABCB11*: ATP binding cassette subfamily B member 11; *ABCC1*: ATP binding cassette subfamily C member 1; *ABCC2*: ATP binding cassette subfamily C member 2; *ABCC3*: ATP binding cassette subfamily C member 3; *ABCC4*: ATP binding cassette subfamily C member 4; *ABCG2*: ATP binding cassette subfamily G member 2; *ACE*: angiotensin I converting enzyme; *ALDH1A1*: aldehyde dehydrogenase 1 family member A1; *APOA1*: apolipoprotein A-I; *APOA5*: apolipoprotein A-V; *APOB*: apolipoprotein B; *APOC3*: apolipoprotein C-III; *APOE*: apolipoprotein E; *CBS*: cystathionine-beta-synthase; *CETP*: cholesteryl ester transfer protein, plasma; *CRP*: C-reactive protein, pentraxin-related; *CYP1A1*: cytochrome P450 family 1 subfamily A member 1; *CYP1A2*: cytochrome P450 family 1 subfamily A member 2; *CYP2B6*: cytochrome P450 family 2 subfamily B member 6; *CYP2C19*: cytochrome P450 family 2 subfamily C member 19; *CYP2C8*: cytochrome P450 family 2 subfamily C member 8; *CYP2C9*: cytochrome P450 family 2 subfamily C member 9; *CYP2D6*: cytochrome P450 family 2 subfamily D member 6; *CYP2E1*: cytochrome P450 family 2 subfamily E member 1; *CYP3A4*: cytochrome P450 family 3 subfamily A member 4; *CYP3A5*: cytochrome P450 family 3 subfamily A member 4; *CYP7A1*: cytochrome P450 family 7 subfamily A member 1; *CYP11B2*: cytochrome P450 family 11 subfamily B member 2; *ESR1*: estrogen receptor 1; *F2*: coagulation factor II, thrombin; *FGB*: fibrinogen beta chain; *GNB3*: guanine nucleotide binding protein (G protein), beta polypeptide 3; HMG-CoA: 3-hydroxy-3-methylglutaryl coenzyme A; *HMGCR*: 3-hydroxy-3-methylglutaryl-CoA reductase; *HTR3B*: 5-hydroxytryptamine (serotonin) receptor 3B, ionotropic*; IL1B*: interleukin 1 beta*; IL6*: interleukin 6; *IL10*: interleukin 10; *ITGB3*: integrin subunit beta 3; *KCNH2*: potassium voltage-gated channel subfamily H member 2; *LDLR*: low density lipoprotein receptor; *LEP*: leptin; *LIPC*: lipase C, hepatic; *LPL*: lipoprotein lipase; *MMP2*: matrix metallopeptidase 2; *MMP3*: matrix metallopeptidase 3; *MTHFR*: methylenetetrahydrofolate reductase (NAD(P)H); *MTTP*: microsomal triglyceride transfer protein; *NOS3*: nitric oxide synthase 3; *NR1I2*: nuclear receptor subfamily 1 group I member 2; *NR1I3*: nuclear receptor subfamily 1 group I member 3; *PON1*: paraoxonase 1; *POR*: P450 (cytochrome) oxidoreductase; *PPARD*: peroxisome proliferator activated receptor delta; *PPARG*: peroxisome proliferator activated receptor gamma; *PRNP*: prion protein; *SLC10A1*: solute carrier family 10 (sodium/bile acid cotransporter), member 1; *SLC15A1*: solute carrier family 15 (oligopeptide transporter), member 1; *SLC22A8*: solute carrier family 22 (organic anion transporter), member 8; *SLCO1A2*: solute carrier organic anion transporter family member 1A2; *SLCO1B1*: solute carrier organic anion transporter family member 1B1; *SLCO1B3*: solute carrier organic anion transporter family member 1B3; *SLCO2B1*: solute carrier organic anion transporter family member 2B1; *TCF20*: transcription factor 20 (AR1); *TNF*: tumor necrosis factor; *TP53*: tumor protein p53*; UGT1A3*: UDP glucuronosyltransferase 1 family, polypeptide A3; *UGT2B7*: UDP glucuronosyltransferase 2 family, polypeptide B7; *USP5*: ubiquitin specific peptidase 5 (isopeptidase T); *VCAM1*: vascular cell adhesion molecule 1.

**Table 2:** Pharmacological profile and pharmacogenetics of angiotensin-converting enzyme (ACE) inhibitors and angiotensin II receptor antagonists.

Hypotensive Agents		
Renin-Angiotensin-Aldosterone System Inhibitors		
**Angiotensin-Converting Enzyme Inhibitors**		
**Drug**	**Properties**	**Pharmacogenetics**
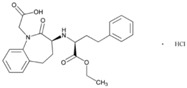	**Name: Benazepril Hydrochloride** **IUPAC Name:** 1*H*-1-Benzazepine-1-acetic acid, 3-[[1-(ethoxycarbonyl)-3-phenylpropyl]amino]-2,3,4,5-tetrahydro-2-oxo-, monohydrochloride, [*S-*(R*,R*)]- **Molecular Formula:** C_24_H_28_N_2_O_5_·HCl **Molecular Weight:** 460.95 g/mol **Mechanism:** Competitive inhibition of ACE activity, which regulates the conversion of angiotensin I to angiotensin II, a potent vasoconstrictor, with resultant lower levels of angiotensin II which causes an increase in plasma renin activity and a reduction in aldosterone secretion. **Effect:** Angiotensin-Converting Enzyme Inhibition.	**Mechanistic genes:** *ACE*, *ACE2*, *ADD1*, *ADRB2*, *AGT*, *AGTR1*, *MTHFR*, *MTR* **Metabolic genes** **Substrate:** *CYP11B2* **Transporter genes:** *SLC15A1*, *SLC15A2*
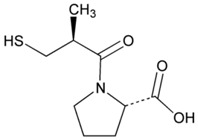	**Name: Captopril** **IUPAC Name:** l-Proline, 1-[(2*S*)-3-mercapto-2-methyl-1-oxopropyl]- **Molecular Formula:** C_9_H_15_NO_3_S **Molecular Weight:** 217.29 g/mol **Mechanism:** Competitive inhibitor of angiotensin-converting enzyme (ACE). Prevents conversion of angiotensin I to angiotensin II, a potent vasoconstrictor, resulting in lower levels of angiotensin II, which causes an increase in plasma renin activity and a reduction in aldosterone secretion. By decreasing local angiotensin II production, ACE inhibitors may decrease vascular tone by reducing direct angiotensin II-induced vasoconstriction and/or angiotensin II-induced increases in sympathetic activity. In hypertensive patients, captopril reduces blood pressure by decreasing total peripheral resistance with no change or an increase in heart rate, stroke volume, or cardiac output (these effects are independent of pre-treatment blood pressure/cardiac output). Causes arterial and possibly venous dilation. In patients with congestive heart failure, captopril decreases total peripheral resistance, pulmonary vascular resistance, pulmonary capillary wedge pressure, and mean arterial and right atrial pressures (cardiac index, cardiac output, stroke volume, and exercise tolerance are increased; heart rate decreases or is unchanged). The drug may also cause regional redistribution of blood flow, principally increasing renal blood flow (glomerular filtration rate is usually unchanged) with slight or no increase in flow in the forearm or hepatic vasculature, respectively. The hypotensive effect of captopril persists longer than inhibition of ACE in blood (unknown whether ACE is inhibited longer in vascular endothelium than in blood). Captopril alone is apparently more effective in reducing blood pressure in high or normal renin hypertension. Serum prolactin concentration has been reported to increase during captopril therapy. **Effect:** Angiotensin-Converting Enzyme Inhibition. Antihypertensive Agent.	**Mechanistic genes:** *ACE*, *ACE2*, *AGT*, *ALB*, *BDKRB2*, *CHRNA2*, *LTA4H*, *MMP2*, *MMP9*, *NOS3*, *REN* **Metabolic genes** **Substrate:** *CYP2D6*, *CYP3A4*, *CYP3A5*, *CYP11B2* **Transporter genes:** *ABCB1*, *SLC15A1*, *SLC22A6*
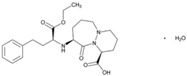	**Name: Cilazapril** **IUPAC Name:** 6*H*-Pyridazino[1,2-*a*][1,2]diazepine-1-carboxylic acid, 9-[[1-(ethoxycarbonyl)-3-phenylpropyl]amino]octahydro-10-oxo-, monohydrate, [1*S*-[1α,9α(R*)]]- **Molecular Formula:** C_22_H_31_N_3_O_5_·H_2_O **Molecular Weight:** 435.51 g/mol **Mechanism:** Competitive inhibitor of angiotensin-converting enzyme. Prevents conversion of angiotensin I to angiotensin II, a potent vasoconstrictor, resulting in lower levels of angiotensin II, and thus causing an increase in plasma renin activity and a reduction in aldosterone secretion. **Effect:** Renin-Angiotensin-Aldosterone System Inhibition; Angiotensin-Converting Enzyme Inhibition.	**Mechanistic genes:** *ACE* **Transporter genes:** *ABCB1*, *SLC15A1*, *SLC15A2*
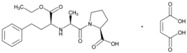	**Name: Enalapril Maleate** **IUPAC Name:** l-Proline, 1-[*N*-[1-(ethoxycarbonyl)-3-phenylpropyl]-l-alanyl]-, (*S*)-, (*Z*)-2-butenedioate (1:1) **Molecular Formula:** C_20_H_28_N_2_O_5_·C_4_H_4_O_4_ **Molecular Weight:** 492.52 g/mol **Mechanism:** Competitive inhibitor of angiotensin-converting enzyme (ACE). Prevents conversion of angiotensin I to angiotensin II, a potent vasoconstrictor. Results in lower levels of angiotensin II, which causes an increase in plasma renin activity and a reduction in aldosterone secretion. **Effect:** Angiotensin-Converting Enzyme Inhibition.	**Mechanistic genes:** *ACE*, *ADRB2*, *AGT*, *AGTR1*, *BDKRB2*, *NOS3* **Metabolic genes** **Substrate:** *CYP3A4*, *CYP3A5* **Transporter genes:** *SLC15A1*, *SLC22A6*, *SLC22A7*, *SLC22A8*, *SLCO1A2* **Pleiotropic genes:** *IL6*
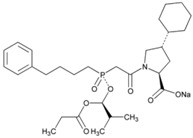	**Name: Fosinopril Sodium** **IUPAC Name:** l-Proline, 4-cyclohexyl-1-[[[2-methyl-1-(1-oxopropoxy)propoxy](4-phenylbutyl)phosphinyl]acetyl]-, sodium salt, [1[S*(R*)],2*a*,4*b*]- **Molecular Formula:** C_30_H_45_NNaO_7_P **Molecular Weight:** 585.64 g/mol **Mechanism:** Competitive inhibitor of angiotensin-converting enzyme. Prevents conversion of angiotensin I to angiotensin II, a potent vasoconstrictor. Results in lower levels of angiotensin II, which causes increase in plasma renin activity and reduction in aldosterone secretion. Vasoactive kallikreins may be decreased in conversion to active hormones by ACEIs, thus reducing blood pressure. **Effect:** Angiotensin-Converting Enzyme Inhibition.	**Mechanistic genes:** *ACE*, *AGT*, *AGTR1*, *BDKRB2*, *NOS3*, *PDE1C* **Transporter genes:** *SLC15A1*, *SLC15A2*
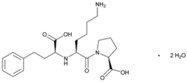	**Name: Lisinopril** **IUPAC Name:** l-Proline, 1-[*N*^2^-(1-carboxy-3-phenylpropyl)-l-lysyl]-, dihydrate, (*S*)- **Molecular Formula:** C_21_H_31_N_3_O_5_·2H_2_O **Molecular Weight:** g/mol **Mechanism:** Competitive inhibitor of angiotensin-converting enzyme (ACE). Prevents conversion of angiotensin I to angiotensin II, a potent vasoconstrictor. **Effect:** Angiotensin-Converting Enzyme Inhibition.	**Mechanistic genes:** *ACE*, *ACE2*, *ADD1*, *AGT*, *AGTR1*, *BDKRB2*, *MMP3*, *NOS3*, *NPPA* **Metabolic genes** **Substrate:** *CYP3A4*, *CYP3A5* **Transporter genes:** *ABCB1*
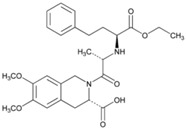	**Name: Moexipril Hydrochloride** **IUPAC Name:** 3-Isoquinolinecarboxylic acid, 2-[2-[[1-(ethoxycarbonyl)-3-phenylpropyl]amino]-1-oxopropyl]-1,2,3,4-tetrahydro-6,7-dimethoxy-, monohydrochloride, [3*S*-[2[R*(R*)],3R*]]- **Molecular Formula:** C_27_H_34_N_2_O_7_·HCl **Molecular Weight:** 535.03 g/mol **Mechanism:** Prevents conversion of angiotensin I to angiotensin II, potent vasoconstrictor, resulting in lower levels of angiotensin II; this causes increase in plasma renin activity and reduction in aldosterone secretion. **Effect:** Angiotensin-Converting Enzyme Inhibition.	**Mechanistic genes:** *ACE*, *ACE2*, *AGT* **Metabolic genes** **Substrate:** *CES1* **Transporter genes:** *SLC15A1*, *SLC15A2*
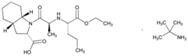	**Name: Perindopril Erbumine** **IUPAC Name:** 1*H*-Indole-2-carboxylic acid, 1-[2-[[1-(ethoxycarbonyl)butyl]amino]-1-oxopropyl]octahydro-, [2*S*-[1[R*(R*)],2α,3*a*β,7*a*β]]-, compd. with 2-methyl-2-propanamine (1:1) **Molecular Formula:** C_19_H_32_N_2_O_5_·C_4_H_11_N **Molecular Weight:** 441.60 g/mol **Mechanism:** A prodrug for perindoprilat, which acts as competitive inhibitor of angiotensin-converting enzyme. Prevents conversion of angiotensin I to angiotensin II, a potent vasoconstrictor, and causes increase in plasma renin activity and reduction in aldosterone secretion. **Effect:** Angiotensin-Converting Enzyme Inhibition.	**Mechanistic genes:** *ACE*, *AGT*, *AGTR1*, *BCHE*, *MMP2*, *TGFB1*, *SFRP4* **Transporter genes:** *ABCB1*, *SLC15A1*, *SLC15A2*
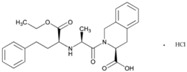	**Name: Quinapril Hydrochloride** **IUPAC Name:** 3-Isoquinolinecarboxylic acid, 2-[2-[[1-(ethoxycarbonyl)-3-phenylpropyl]amino]-1-oxopropyl]-1,2,3,4-tetrahydro-, monohydrochloride, [3*S*-[2[R*(R*)],3R*]] **Molecular Formula:** C_25_H_30_N_2_O_5_ **Molecular Weight:** 474.98 g/mol **Mechanism:** Prodrug, not pharmacologically active until hydrolyzed in liver to quinaprilat. Competitive inhibitor of ACE. Prevents conversion of angiotensin I to angiotensin II, a potent vasoconstrictor. Lower levels of angiotensin II cause increase in plasma renin activity and reduction in aldosterone secretion. A CNS mechanism may also be involved in hypotensive effect as angiotensin II increases adrenergic outflow from CNS. Vasoactive kallikreins may be decreased in conversion to active hormones by ACEIs, thus reducing blood pressure. **Effect:** Angiotensin-Converting Enzyme Inhibition.	**Mechanistic genes:** *ACE*, *AGT*, *AGTR1*, *BDKBR2*, *NR1I2*, *TGFB1* **Metabolic genes** **Substrate:** *CYP11B2* **Transporter genes:** *SLC15A1*, *SLC15A2*
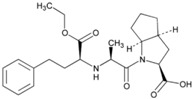	**Name: Ramipril** **IUPAC Name:** Cyclopenta[*b*]pyrrole-2-carboxylic acid, 1-[2-[[1-(ethoxycarbonyl)-3-phenylpropyl]amino]-1-oxopropyl]octahydro-, [2*S*-[1[R*(R*)],2α,3*a*β,6*ab*]]- **Molecular Formula:** C_23_H_32_N_2_O_5_ **Molecular Weight:** 416.51 g/mol **Mechanism:** Ramipril undergoes enzymatic saponification by esterases in liver, to its active metabolite ramiprilat. Ramiprilat reversibly binds to angiotensin-converting enzyme, thus preventing formation of potent vasoconstrictor angiotensin II from angiotensin I. CNS mechanism may also be involved in hypotensive effect, as angiotensin II increases adrenergic outflow from CNS. Vasoactive kallikreins may be decreased in conversion to active hormones by ACEIs, thus reducing blood pressure. **Effect:** Angiotensin-Converting Enzyme Inhibition.	**Mechanistic genes:** *ACE*, *AGT*, *AGTR1*, *BCHE*, *BDKRB2*, *COL1A1*, *MMP2*, *NOS3*, *REN*, *TGBFB1* **Metabolic genes** **Substrate:** *CYP11B2* **Pleiotropic genes:** *APOE* **Transporter genes:** *SLC15A1*, *SLC15A2*
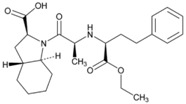	**Name: Trandolapril** **IUPAC Name:** (2*S*,3*aR*,7*aS*)-1-[(*S*)-*N*-[(*S*)-1-Carboxy-3-phenylpropyl]alanyl]hexahydro-2-indolinecarboxylic acid, 1-ethyl ester **Molecular Formula:** C_24_H_34_N_2_O_5_ **Molecular Weight:** 430.54 g/mol **Mechanism:** Prevents formation of angiotensin II from angiotensin I. Trandolapril must undergo enzymatic hydrolysis, mainly in liver, to its biologically active metabolite, trandolaprilat. A CNS mechanism may also be involved in hypotensive effect as angiotensin II increases adrenergic outflow from CNS. Vasoactive kallikreins may be decreased in conversion to active hormones by ACEIs, thus reducing blood pressure. **Effect:** Angiotensin-Converting Enzyme Inhibition.	**Mechanistic genes:** *ACE*, *ADD1* **Metabolic genes** **Substrate:** *CES1* **Transporter genes:** *SLC15A1*, *SLC15A2*
**Angiotensin II Receptor Antagonists**		
**Drug**	**Properties**	**Pharmacogenetics**
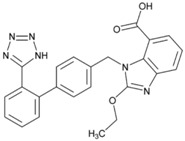	**Name: Candesartan Cilexetil** **IUPAC Name:** 1*H*-Benzimidazole-7-carboxylic acid, 2-ethoxy-1-[[2′-(1*H*-tetrazol-5-yl)[1,1′-biphenyl]-4-yl]methyl]- **Molecular Formula:** C_24_H_20_N_6_O_3_ **Molecular Weight:** 440.45 g/mol **Mechanism:** Candesartan is an angiotensin receptor antagonist, blocking vasoconstriction and the aldosterone-secreting effects (reabsorption of sodium and water) of angiotensin II. **Effect:** Angiotensin II Receptor Antagonists.	**Mechanistic genes:** *ACE*, *AGT*, *AGTR1*, *BDKRB2*, *NOS3*, *PTGS1*, *TGFB1* **Metabolic genes** **Substrate:** *CYP1A1*, *CYP2C8*, *CYP2C9*, *CYP11B2*, *UGT1A3*, *UGT1A5*, *UGT2B7* **Transporter genes:** *ABCB1*, *ABCG2*
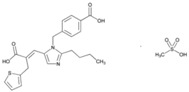	**Name: Eprosartan Mesylate** **IUPAC Name:** 2-Thiophenepropanoic acid, α-[[2-butyl-1-[(4-carboxyphenyl)methyl]-1*H*-imidazol-5-yl]methylene]-, (*E*)-, monomethanesulfonate **Molecular Formula:** C_23_H_24_N_2_O_4_S·CH_4_O_3_S **Molecular Weight:** 520.62 g/mol **Mechanism:** A nonbiphenyl, nontetrazole angiotensin II receptor (AT1) antagonist. Blocks the vasoconstrictor and aldosterone-secreting effects of angiotensin II by selectively blocking the binding of angiotensin II to the AT1 receptor in many tissues, such as vascular smooth muscle and adrenal gland. Does not bind to or block other hormone receptors or ion channels known to be important in cardiovascular regulation. **Effect:** Angiotensin II Receptor Antagonists.	**Mechanistic genes:** *ACE*, *AGTR1* **Metabolic genes** **Substrate:** *CYP2C9* **Transporter genes:** *ABCB1*, *ABCC2*, *ABCG2*
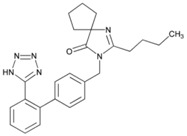	**Name: Irbesartan** **IUPAC Name:** 1,3-Diazaspiro[4.4]non-1-en-4-one, 2-butyl-3-[[2′-(1*H*-tetrazol-5-yl)[1,1′-biphenyl]-4-yl]methyl]- **Molecular Formula:** C_25_H_28_N_6_O **Molecular Weight:** 428.53 g/mol **Mechanism:** Irbesartan binds to AT1 angiotensin II receptor. This binding prevents angiotensin II from binding to receptor, thereby blocking the vasoconstriction and aldosterone-secreting effects of angiotensin II. **Effect:** Angiotensin II Receptor Antagonists.	**Mechanistic genes:** *ADRA1A*, *AGT*, *AGTR1*, *APOB*, *BDKRB2*, *JUN*, *LDLR*, *NOS3*, *PTGS1*, *TGFB1* **Metabolic genes** **Substrate:** *CYP1A2*, *CYP2C8*, *CYP2C9*, *CYP2D6*, *CYP3A4*, *CYP3A5*, *CYP11B2*, *UGT1A3* **Transporter genes:** *ABCB1 ABCG2* **Pleiotropic genes:** *APOE*
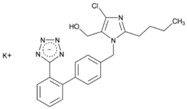	**Name: Losartan Potassium** **IUPAC Name:** 1*H*-Imidazole-5-methanol, 2-butyl-4-chloro-1-[[2′-(1*H*-tetrazol-5-yl)[1,1′-biphenyl]-4-yl]methyl]-, monopotassium salt **Molecular Formula:** C_22_H_22_ClKN_6_O **Molecular Weight:** 461.00 g/mol **Mechanism:** As a selective and competitive nonpeptide angiotensin II receptor antagonist, losartan blocks vasoconstrictor and aldosterone-secreting effects of angiotensin II. Losartan increases urinary flow rate and, in addition to being natriuretic and kaliuretic, increases excretion of chloride, magnesium, uric acid, calcium, and phosphate. **Effect:** Angiotensin II Receptor Antagonists.	**Mechanistic genes:** *ACE*, *ADD1*, *AGT*, *AGTR1*, *AGTR2*, *ALB*, *BDKRB2*, *EDN1*, *FOS*, *MMP2*, *NOS3*, *PDGFRB*, *TGFB1* **Metabolic genes** **Substrate:** *CYP1A2*, *CYP2C8*, *CYP2C9*, *CYP2C19*, *CYP3A4*, *CYP3A5*, *CYP11B2*, *UGT1A1*, *UGT1A3*, *UGT1A10*, *UGT2B7*, *UGT2B17* **Transporter genes:** *ABCB1*, *ABCG2*, *SLC2A9*, *SLC22A6*, *SLC22A12* **Pleiotropic genes:** *TNF*
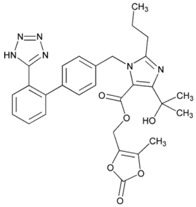	**Name:** Olmesartan Medoxomil **IUPAC Name:** 1*H*-Imidazole-5-carboxylic acid, 4-(1-hydroxy-1-methylethyl)-2-propyl-1-[[2′-(1H-tetrazol-5-yl) [1,1′-biphenyl]-4-yl]methyl]-, (5-methyl-2-oxo-1,3-dioxol-4-yl)methyl ester **Molecular Formula:** C_29_H_30_N_6_O_6_ **Molecular Weight:** 558.59 g/mol **Mechanism:** Blocks vasoconstrictor and aldosterone-secreting effects of angiotensin II. Interacts reversibly at AT1 and AT2 receptors and has slow dissociation kinetics (has greater affinity for AT1 receptor). Olmesartan increases urinary flow rate and, besides being natriuretic and kaliuretic, increases excretion of chloride, magnesium, uric acid, calcium, and phosphate. **Effect:** Angiotensin II Receptor Antagonists.	**Mechanistic genes:** *AGTR1*, *ACE2*, *EDN1*, *TGFB1* **Metabolic genes** **Substrate:** *CMBL*, *CYP2C9* **Transporter genes:** *ABCB1*, *ABCC2*, *ABCG2*, *SLCO1B1*, *SLCO1B3*, *SLCO1A2*, *SLC22A8* **Pleiotropic genes:** *APOE*
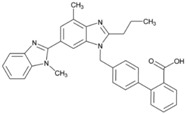	**Name:** Telmisartan **IUPAC Name:** [1,1′-Biphenyl]-2-carboxylic acid, 4′-[(1,4′-dimethyl-2′-propyl[2,6′-bi-1H-benzimidazol]-1′-yl)methyl]- **Molecular Formula:** C_33_H_30_N_4_O_2_ **Molecular Weight:** 514.62 g/mol **Mechanism:** A nonpeptide AT_1_ angiotensin II receptor antagonist. This binding prevents angiotensin II from binding to receptor thereby blocking vasoconstriction and aldosterone-secreting effects of angiotensin II. **Effect:** Angiotensin II Receptor Antagonists.	**Mechanistic genes:** *ACE*, *AGT*, *AGTR1*, *BDKRB2*, *ERAP1*, *PPARG* **Metabolic genes** **Substrate:** *CYP2C9*, *CYP2C19*, *CYP11B2*, *UGT1A1* **Transporter genes:** *ABCB1*, *ABCC2*, *ABCG2*
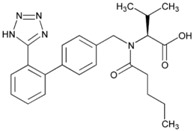	**Name: Valsartan** **IUPAC Name:** l-Valine, *N*-(1-oxopentyl)-*N*-[[2′-(1*H*-tetrazol-5-yl)[1,1′-biphenyl]-4-yl]methyl]- **Molecular Formula:** C_24_H_29_N_5_O_3_ **Molecular Weight:** 435.52 g/mol **Mechanism:** Displaces angiotensin II from AT_1_ receptor and produces its blood pressure-lowering effects by antagonizing AT_1_-induced vasoconstriction, aldosterone release, catecholamine release, arginine vasopressin release, water intake, and hypertrophic responses. **Effect:** Angiotensin II Receptor Antagonists.	**Mechanistic genes:** *ACE*, *AGT*, *AGTR1*, *ALB*, *BDKRB2*, *ERAP1*, *GNB3*, **Metabolic genes** **Substrate:** *CYP2C9*, *CYP2C19*, *CYP2D6*, *CYP3A4*, *CYP3A5*, *CYP11B2* **Transporter genes:** *ABCC2*, *SLCO1B1*, *SLCO1B3*

*ABCB1*: ATP binding cassette subfamily B member 1, *ABCB2*: ATP binding cassette subfamily B member 2, *ABCB10*: ATP binding cassette subfamily B member 10, *ABCB11*: ATP binding cassette subfamily B member 11, *ABCC1*: ATP-binding cassette, sub-family C (CFTR/MRP), member 1, *ABCC2*: ATP-binding cassette, sub-family C (CFTR/MRP), member 2, *ABCC3*: ATP binding cassette subfamily C (CFTR/MRP), member 3, *ABCC4*: ATP binding cassette subfamily C (CFTR/MRP), member 4, *ABCC5*: ATP binding cassette subfamily C (CFTR/MRP), member 5, *ABCC8*: ATP-binding cassette, sub-family C (CFTR/MRP), member 8, *ABCC9*: ATP-binding cassette, sub-family C (CFTR/MRP), member 9, *ABCC10*: ATP binding cassette subfamily C (CFTR/MRP), member 10, *ABCG2*: ATP binding cassette subfamily G member 2, *ACCNs*: Acid-Sensing Ion Channels, *ACE*: angiotensin I converting enzyme (peptidyl-dipeptidase A) 1, *ACE2*: angiotensin I converting enzyme 2, *ADA*: adenosine deaminase, *ADD1*: adducin 1 (alpha), *ADORA1*: adenosine receptor A1, *ADORA2A*: adenosine receptor A2A, *ADORA2B*: adenosine receptor A2B, *ADRA1A*: adrenoceptor alpha 1A, *ADRA1B*: adrenoceptor alpha 1B, *ADRA1D*: adrenoceptor alpha 1D, *ADRA2A*: adrenoceptor alpha 2A, *ADRA2B*: adrenoceptor alpha 2B, *ADRA2C*: adrenoceptor alpha 2C, *ADRB1*: adrenergic, beta-1-, receptor, *ADRB2*: adrenergic, beta-2-, receptor, surface, *AGPAT2*: 1-acylglycerol-3-phosphate O-acyltransferase 2 (lysophosphatidic acid acyltransferase, beta), *AGT*: angiotensinogen (serpin peptidase inhibitor, clade A, member 8), *AGTR1*: angiotensin II receptor type 1, *AHR*: aryl hydrocarbon receptor, *AKR1C4*: aldo-keto reductase family 1, member C4 (chlordecone reductase; 3-alpha hydroxysteroid dehydrogenase, type I; dihydrodiol dehydrogenase 4), *ALB*: albumin, *ALDH2*: aldehyde dehydrogenase 2 family (mitochondrial), *ALOX5*: arachidonate 5-lipoxygenase, *AOC3*: amine oxidase, copper containing 3, *APC*: adenomatous polyposis coli, *APOB*: apolipoprotein B, *APOE*: apolipoprotein E, *APP*: amyloid beta (A4) precursor protein, *AR*: androgen receptor, *ARG1*: arginase, liver, *ASIC1*: acid sensing ion channel subunit 1, *ASIC2*: acid sensing ion channel subunit 2, *ATP1A1*: ATPase Na^+^/K^+^ transporting subunit alpha 1, *AVPR1A*: arginine vasopressin receptor 1A, *AVPR2*: arginine vasopressin receptor 2, *BDKRB2*: bradykinin receptor B2, *BIRC5 (NAIP)*: NLR family apoptosis inhibitory protein, *CA1*: carbonic anhydrase 1, *CA2*: carbonic anhydrase 2, *CA4*: carbonic anhydrase 4, *CA9*: carbonic anhydrase 9, *CA12*: carbonic anhydrase 12, *CACNA1A*: calcium channel, voltage-dependent, alpha-1 subunit, *CACNA1B*: calcium channel, voltage-dependent, beta-1 subunit, *CACNA1C*: calcium channel, voltage-dependent, alpha-1 subunit, *CACNA1D*: calcium channel, voltage-dependent, delta-1 subunit, *CACNA1F*: calcium channel, voltage-dependent subunit alpha1 F, *CACNA1G*: calcium channel, voltage-dependent subunit alpha1 G, *CACNA1I*: calcium channel, voltage-dependent subunit alpha1 I, *CACNA1S*: calcium channel, voltage-dependent subunit alpha1 S, *CACNA2D1*: calcium voltage-gated channel auxiliary subunit alpha 2 delta 1, *CACNB1*: calcium voltage-gated channel auxiliary subunit beta 1, *CACNB2*: calcium voltage-gated channel auxiliary subunit beta 2, *CACNB3*: calcium voltage-gated channel auxiliary subunit beta 3, *CACNB4*: calcium voltage-gated channel auxiliary subunit beta 4, *CACNG1*: calcium voltage-gated channel auxiliary subunit gamma 1, *CALM1*: calmodulin 1, *CCL23*: C-C motif chemokine ligand 23, *CEL*: carboxyl ester lipase, *CES1*: carboxylesterase 1, *CFH*: complement factor H, *CFTR*: cystic fibrosis transmembrane conductance regulator (ATP-binding cassette sub-family C, member 7), *CHAT*: choline acetyltransferase, *CHRM1*: cholinergic receptor muscarinic 1, *CHRM2*: cholinergic receptor muscarinic 2, *CHRM3*: cholinergic receptor muscarinic 3, *CHRM4*: cholinergic receptor muscarinic 4, *CHRM5*: cholinergic receptor muscarinic 5, *CHRNA1*: cholinergic receptor, nicotinic, alpha 1, *CHRNA2*: cholinergic receptor, nicotinic, alpha 2, *CHRNA4*: cholinergic receptor, nicotinic, alpha 4, *CHRNA7*: cholinergic receptor, nicotinic, alpha 7, *CHRNB2*: cholinergic receptor, nicotinic, beta 2, *CMBL*: carboxymethylenebutenolidase homolog, *COL1A1*: collagen, type I, alpha 1, *COMT*: catechol-O-methyltransferase, *CPNE1*: copine 1, *CTNNB1*: catenin beta 1, *CYP1A1*: cytochrome P450, family 1, subfamily A, polypeptide 1, *CYP1A2*: cytochrome P450 family 1 subfamily A member 2, *CYP1B1*: cytochrome P450 family 1 subfamily B member 1, *CYP2C8*: cytochrome P450, family 2, subfamily C, polypeptide 8, *CYP2C9*: cytochrome P450, family 2, subfamily C, polypeptide 9, *CYP2D6*: cytochrome P450, family 2, subfamily D, polypeptide 6, *CYP2E1*: cytochrome P450, family 2, subfamily E, polypeptide 1, *CYP2J2*: cytochrome P450, family 2, subfamily J, polypeptide 2, *CYP3A4*: cytochrome P450 family 3 subfamily A member 4, *CYP3A5*: cytochrome P450 family 3 subfamily A member 5, *CYP3A7*: cytochrome P450 family 3 subfamily A member 7, *CYP7A1*: cytochrome P450, family 7, subfamily A, polypeptide 1, *CYP11B1*: cytochrome P450, family 11, subfamily B, polypeptide 1, *CYP11B2*: cytochrome P450, family 11, subfamily B, polypeptide 2, *CYP17A1*: cytochrome P450, family 17, subfamily A, polypeptide 1, *CYP19A1*: cytochrome P450, family 19, subfamily A, polypeptide 1, *CYPs*: cytochrome P450 family, *DDC*: dopa decarboxylase, *DRD1*: dopamine receptor D1, *DRD2*: dopamine receptor D2, *DRD5*: dopamine receptor D5, *EDN1*: endothelin 1, *EDNRA*: endothelin receptor type A, *EDNRB*: endothelin receptor type B, *ERAP1*: endoplasmic reticulum aminopeptidase 1, *ERBB2*: v-erb-b2 erythroblastic leukemia viral oncogene homolog 2, neuro/glioblastoma derived oncogene homolog (avian), *ESR1*: estrogen receptor 1, *FOS*: FBJ murine osteosarcoma viral oncogene homolog, *GLUL*: glutamine synthetase, *GNB3*: guanine nucleotide binding protein (G protein), beta polypeptide 3, *GSTA1*: glutathione *S-*transferase alpha 1, *GSTA2*: glutathione *S-*transferase alpha 2, *GSTM1*: glutathione *S-*transferase mu 1, *GSTP1*: glutathione *S-*transferase pi 1, *GSTs*: glutathione *S-*transferases, *GSTT1*: glutathione *S-*transferase theta 1, *GUCY1A2*: guanylate cyclase 1 soluble subunit alpha 2, *HBB*: hemoglobin, beta, *HDAC2*: histone deacetylase 2, *HFE*: hemochromatosis, *HIF1A*: hypoxia inducible factor 1 alpha subunit, *HLA-A*: major histocompatibility complex, class I, A, *HLA-B*: major histocompatibility complex, class I, B, *HM13*: histocompatibility minor 13,*HNF4*: hepatocyte nuclear factor 4, alpha, *HRH1*: histamine receptor H1, *HRH2*: histamine receptor H2, *IDO1*: indoleamine 2,3-dioxygenase 1, *IL10*: interleukin 10, *IL1B*: interleukin 1, beta, *IL6*: interleukin 6 (interferon, beta 2), *JUN*: Jun proto-oncogene, AP-1 transcription factor subunit, *KCNE1*: potassium voltage-gated channel subfamily E member 1, *KCNH2*: potassium voltage-gated channel subfamily H member 2, *KCNJ1*: potassium voltage-gated channel subfamily J member 1, *KCNJ11*: potassium inwardly-rectifying channel, subfamily J, member 11, *KCNJ5*: potassium voltage-gated channel subfamily J member 5, *KCNMA1*: potassium calcium-activated channel subfamily M alpha 1, *KCNQ1*: potassium inwardly-rectifying channel, subfamily Q, member 1, *KDR*: kinase insert domain receptor (a type III receptor tyrosine kinase), *LEF1*: lymphoid enhancer binding factor 1, *LDLR*: low density lipoprotein receptor, *LTA4H*: leukotriene A4 hydrolase,*MAOA*: monoamine oxidase A, *MAOB*: monoamine oxidase B, *MGMT*: *O*-6-methylguanine-DNA methyltransferase, *MMP2*: matrix metallopeptidase 2, *MMP3*: matrix metallopeptidase 3, *MMP9*: matrix metallopeptidase 9, *MTHFR*: methylenetetrahydrofolate reductase, *MT1A*: metallothionein 1A, *mtlD*: mannitol-1-phosphate dehydrogenase, NAD-dependent, *MTR*: 5-methyltetrahydrofolate-homocysteine methyltransferase, *NAT2*: *N*-acetyltransferase 2 (arylamine *N*-acetyltransferase), *NOMO1*: NOMO nodal modulator 1, *NOS3*: nitric oxide synthase 3 (endothelial cell), *NPPA*: natriuretic peptide precursor A, *NPR1*: natriuretic receptor precursor 1, *NR1I2*: nuclear receptor subfamily 1, group I, member 2, *NR3C1*: nuclear receptor subfamily 3, group C, member 1 (glucocorticoid receptor), *NR3C2*: nuclear receptor subfamily 3, group C, member 2 (glucocorticoid receptor), *P2RY12*: purinergic receptor P2Y, G-protein coupled, 12, *ORM1*: orosomucoid 1, *P4HA1*: prolyl 4-hydroxylase subunit alpha 1, *PARP1*: poly(ADP-ribose) polymerase 1, *PDE1C*: phosphodiesterase 1C, *PDE1A*: phosphodiesterase 1A, *PDE3A*: phosphodiesterase 3A, *PDE1B*: phosphodiesterase 1B, *PDE4A*: phosphodiesterase 4A, *PDE4B*: phosphodiesterase 4B, *PDE5A*: phosphodiesterase 5A, cGMP-specific, *PDE6A*: phosphodiesterase 6A, cGMP-specific, *PDE6G*: phosphodiesterase 6G, *PDE6H*: phosphodiesterase 6H, *PDE10A*: phosphodiesterase 10A, *PDE11A*: phosphodiesterase 11A, *PDGFRB*: platelet-derived growth factor receptor, beta polypeptide, *PGD*: phosphogluconate dehydrogenase, *PGR*: progesterone receptor, *PKD1*: polycystin 1, transient receptor potential channel interacting, *PKD2*: polycystin 2, transient receptor potential channel interacting, *PLAU*: plasminogen activator, urokinase, *PLAT*: plasminogen activator, tissue type, *PPARD*: peroxisome proliferator-activated receptor-delta, *PPARG*: peroxisome proliferator-activated receptor-gamma, *PTGERs*: prostaglandin E receptors, *PTGIR*: prostaglandin I2 (prostacyclin) receptor (IP), *PTGS1*: prostaglandin-endoperoxide synthase 1 (prostaglandin G/H synthase and cyclooxygenase), *PTGS2*: prostaglandin-endoperoxide synthase 2 (prostaglandin G/H synthase and cyclooxygenase), *RCAN1*: regulator of calcineurin 1, *REN*: renin, *RIC3*: RIC3 acetylcholine receptor chaperone, *SCN5A*: sodium voltage-gated channel alpha subunit 5, *SCNN1A*: sodium channel epithelial 1 alpha subunit, *SCNN1B*: sodium channel epithelial 1 beta subunit, *SCNN1D*: sodium channel epithelial 1 delta subunit, *SCNN1G*: sodium channel, nonvoltage-gated 1, gamma, *SCNs*: sodium channels, nonvoltage-gated, *SFRP4*: secreted frizzled related protein 4, *SHBG*: sex hormone binding globulin, *SLC6A2*: solute carrier family 6 (neurotransmitter transporter, noradrenalin), member 2, *SLC6A4*: solute carrier family 6 member 4, *SLC9A1*: solute carrier family 9 member 1, *SLC10A1*: solute carrier family 10 member 1, *SLC12A1*: solute carrier family 12 member 1, *SLC12A2*: solute carrier family 12 member 2, *SLC12A3*: solute carrier family 12, member 3, *SLC12A4*: solute carrier family 12, member 4, *SLC14A1*: solute carrier family 14 member 1, *SLC14A2*: solute carrier family 14 member 2, *SLC15A1*: solute carrier family 15 member 1, *SLC15A2*: solute carrier family 15 member 2, *SLC18A2*: solute carrier family 18, member 2, *SLC19A1*: solute carrier family 19 (folate transporter), member 1, *SLC22A1*: solute carrier family 22 member 1, *SLC22A2*: solute carrier family 22 member 2, *SLC22A3*: solute carrier family 22 member 3, *SLC22A4*: solute carrier family 22 member 4, *SLC22A5*: solute carrier family 22 member 5, *SLC22A6*: solute carrier family 22 member 6, *SLC22A7*: solute carrier family 22 member 7, *SLC22A8*: solute carrier family 22 member 8, *SLC22A11*: solute carrier family 22 member 11, *SLC22A16*: solute carrier family 22 member 16, *SLC29A*: solute carrier family 29, *SLCO1A2*: solute carrier organic anion transporter family, member 1A2, *SLCO1B1*: solute carrier organic anion transporter family, member 1B1, *SLCO1B3*: solute carrier organic anion transporter family, member 1B3, *SRD5A1*: steroid 5 alpha-reductase 1, *SULT1A1*: sulfotransferase family, cytosolic, 1A, phenol-preferring, member 1, *SULT1A3*: sulfotransferase family, cytosolic, 1A, phenol-preferring, member 3, *TGFB1*: transforming growth factor, beta 1, *TNF*: tumor necrosis factor (TNF superfamily, member 2, *TP53*: tumor protein p53, *TTLL3*: tubulin tyrosine ligase like 3, *UGT1A1*: UDP glucuronosyltransferase 1 family, polypeptide A1, *UGT1A3*: UDP glucuronosyltransferase 1 family, polypeptide A3, *UGT1A6*: UDP glucuronosyltransferase 1 family, polypeptide A6, *UGT1A9*: UDP glucuronosyltransferase 1 family, polypeptide A9, *UGT2B7*: UDP glucuronosyltransferase 2 family, polypeptide B7, *UGT2B17*: UDP glucuronosyltransferase 2 family, polypeptide B17, *UGTs*: UDP glucuronosyltransferase family, *USP5*: ubiquitin specific peptidase 5 (isopeptidase T), *WNK1*: WNK lysine deficient protein kinase 1.
